# Navigating uncertainty in maximum body size in marine metazoans

**DOI:** 10.1002/ece3.11506

**Published:** 2024-06-05

**Authors:** Craig R. McClain, Thomas J. Webb, Noel A. Heim, Matthew L. Knope, Pedro M. Monarrez, Jonathan L. Payne

**Affiliations:** ^1^ Department of Biology University of Louisiana at Lafayette Lafayette Louisiana USA; ^2^ Ecology & Evolutionary Biology, School of Biosciences University of Sheffield Sheffield UK; ^3^ Department of Earth and Climate Sciences Tufts University Medford Massachusetts USA; ^4^ Department of Biology University of Hawaiʻi at Hilo Hilo Hawaii USA; ^5^ Department of Earth and Planetary Sciences Stanford University Stanford California USA; ^6^ Department of Earth, Planetary, and Space Sciences University of California, Los Angeles Los Angeles California USA

**Keywords:** body size, macroecology, macroevolution, maximum size, variation

## Abstract

Body size is a fundamental biological trait shaping ecological interactions, evolutionary processes, and our understanding of the structure and dynamics of marine communities on a global scale. Accurately defining a species' body size, despite the ease of measurement, poses significant challenges due to varied methodologies, tool usage, and subjectivity among researchers, resulting in multiple, often discrepant size estimates. These discrepancies, stemming from diverse measurement approaches and inherent variability, could substantially impact the reliability and precision of ecological and evolutionary studies reliant on body size data across extensive species datasets. This study examines the variation in reported maximum body sizes across 69,570 individual measurements of maximum size, ranging from <0.2 μm to >45 m, for 27,271 species of marine metazoans. The research aims to investigate how reported maximum size variations within species relate to organism size, taxonomy, habitat, and the presence of skeletal structures. The investigation particularly focuses on understanding why discrepancies in maximum size estimates arise and their potential implications for broader ecological and evolutionary studies relying on body size data. Variation in reported maximum sizes is zero for 38% of species, and low for most species, although it exceeds two orders of magnitude for some species. The likelihood of zero variation in maximum size decreased with more measurements and increased in larger species, though this varied across phyla and habitats. Pelagic organisms consistently had low maximum size range values, while small species with unspecified habitats had the highest variation. Variations in maximum size within a species were notably smaller than interspecific variation at higher taxonomic levels. Significant variation in maximum size estimates exists within marine species, and partially explained by organism size, taxonomic group, and habitat. Variation in maximum size could be reduced by standardized measurement protocols and improved meta‐data. Despite the variation, egregious errors in published maximum size measurements are rare, and their impact on comparative macroecological and macroevolutionary research is likely minimal.

## Graphical abstract

This research investigates maximum body sizes in 27,581 marine species from 24 phyla globally in the modern oceans. Analyzing size data and factors like taxonomy, habitat, and measurement methods revealed significant variation in reported maximum sizes. Despite variations, only about 5% of species experienced significant errors, emphasizing the need for standardized measurement protocols to minimize discrepancies in maximum size reporting without significantly altering species rankings in macroecological and macroevolutionary studies.

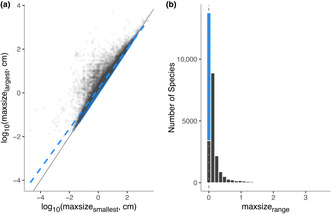

## 1 INTRODUCTION

Body size is a fundamental biological trait and has a long‐history of intensive study across many biological disciplines, including ecology, evolution, physiology, and medicine (Bonner, 2007). Body size can affect an organism's resource use (Brown et al., 2004), level of success in competition (Grant, 1968; Hutchinson, 1959; Wilson, 1975), and interactions with predators and prey (Barnes et al., 2010; Costa, 2009). Differently sized animals may be able to use different resources in the landscape and at different timescales (Cooke et al., 2022; McClain et al., 2006; Ritchie & Olff, 1999). In addition, body size can influence the efficiency of movement, which can be important in determining an animal's ability to disperse, migrate, or hunt (Goldbogen et al., 2012). Body size is subject to natural selection (Nagel & Schluter, 1998; Schluter & Smith, 1986), with different body sizes being favored in different environments (Aava, 2001; Gearty et al., 2018; Gearty & Payne, 2020; Knope & Scales, 2013; Knouft, 2004; Lomolino, 2005; Poulin, 1996). The adaptive importance of body size is also strengthened by the direct link of body size to reproductive success, where larger individuals of a species may have higher reproductive output (Bosch & Vicens, 2006; Wiklund & Kaitala, 1995). Thus, by analyzing body‐size data, scientists can uncover patterns in the structure and dynamics of communities, and make predictions about how organisms may respond to changing environments (Hunt & Roy, 2006; Millien, 2004; Sheridan & Bickford, 2011), and gain a deeper understanding of the evolutionary history of life on Earth (Alroy, 1998; Heim et al., 2015; Payne et al., 2008).

One reason for the popularity of body size as a research topic, aside from its fundamental importance in many biological processes, is that it is relatively easy to measure and straightforward to compare across the tree of life from viruses and archaea to blue whales (Brown, 1995). Due to the ease of measuring body size, large amounts of data exist for many different species, making it a valuable trait for comparative studies and meta‐analyses (Bloom et al., 2018; DeLong et al., 2010; Harmon et al., 2010; Heim et al., 2017; Hillebrand & Azovsky, 2001; Thornton & Fletcher Jr, 2014). Furthermore, the abundance of data on body size allows for detailed analyses of patterns and trends in body size across different ecological, geographical, and evolutionary scales.

Despite the ease of taking body‐size measurements, the accurate characterization of body size, including assigning a single, appropriate value to a species, can be challenging. Multiple estimates of body size often arise for a single species. In part, this situation reflects biologically important ontogenetic and intraspecific variation. However, some of this size variation arises from multiple attempts to characterize the size of a species using a single size metric. For example, “maximum size,” which is quite often a target measurement for both biological and practical reasons, maybe collected by different researchers, at different places or times, making different measurement choices, or subject to other methodological issues. In addittion, many tools (e.g., rulers, calipers, lasers, scanners, scales), and measures (e.g., length, area, volume, mass), exist to quantify body size. Errors can also easily arise, such as those due to the use of inaccurate instruments, measurement variability among observers, and measurement bias due to subjectivity or inappropriate scaling methods, in addition to simple typographical errors that can propagate as data are transcribed from one source to another. For example, the Australian trumpet snail (*Syrinx aruanus*) has a reported maximum length value in the literature, databases, and websites of either 91.4 cm or 72.2 cm (McClain et al., 2015). Further research showed that both of these measurements are attributed to the same specimen and collector and that the larger measurement (91.4 cm) is an error (McClain et al., 2015). In addition, for some taxa, standards on measurement do not exist. For example, for species of wood‐boring bivalves in the families Xylophagiidae and Terenidae, reported length measurements can reflect the shell alone, often millimeters to centimeters in length, or include the siphons that reach a meter in some species (Hanks et al., in review). Thus, multiple estimates of body size for a single species can differ substantially and potentially affect the accuracy and reproducibility of ecological and evolutionary studies that rely on body size data. In studies that address size evolution across hundreds to thousands or tens of thousands of species, it may not be realistic or even possible to vet all size data from other sources to identify and address these sources of error or variability. Moreover, biologists currently lack a comprehensive understanding of what factors may bias the size measurements due to a lack of research.

Here, we examine variability in reported maximum size measurements within species across marine Metazoa. Multiple estimates of maximum size can occur tied to real intraspecific variation coded into the literature as holotypes, paratypes, and neotypes, or reflecting differences in body size varying environmentally and recorded in different regional inventories. For each species in our dataset, we characterize the largest maximum size reported (maxsize_largest_), smallest maximum size reported (maxsize_smallest_), and the difference between the two (maxsize_range_). We analyze maximum size measurements for 27,271 marine species with multiple available estimates of maximum size. These species range in reported total length from the smallest value of maxsize_smallest_ of 0.195 microns (*Batillipes tubernatis*, a benthic tardigrade) to the largest value of maxsize_largest_ of 45.7 meters (*Praya dubia*, the giant siphonophore). We specifically test how ranges in maximum size within marine species varies with: (1) the reported size of the organism, (2) taxonomic group, (3) habitat, and (4) presence of exo‐ or endo‐skeleton. We chose maximum size because it is commonly used in broad‐scale studies of body‐size evolution, often with the justification of avoiding the inclusion of juveniles, providing a consistent approach to species with indeterminate growth (Heim et al., 2015), and difficulty in estimating the entire size distribution within a single species across habitats. We hypothesize the range in (log‐transformed) maximum size estimates is: (1) greater for smaller organisms because of greater error in measurement relative to body size; (2) greater in some taxonomic groups due to either complex bauplans, including coloniality, or measurement standards; (3) greater in pelagic organisms given their often‐gelatinous nature, indeterminate growth, and difficulty in collection; and (4) greater in those organisms lacking hard skeletons, which makes measurement more difficult and variable, leading to greater variation in measured lengths due to the ease of body deformation. Finally, we examine those species with extreme (>2 orders of magnitude) range in maximum size measurements and consider the impacts that errors in maximum size estimates may have on comparative macroecological and macroevolutionary studies that rely on collations of body size data from the literature.

## 2 METHODS

Maximum size as the largest linear dimension was collected for marine metazoans from 356 online databases and published literature. We choose linear dimension (e.g., height, length, width, and diameter), for this study because it is the most commonly reported measure of size in the literature. While mass, rather than length, scales proportionally with energetics and metabolic rate, length scales with mass in higher taxa (Benke et al., 1999; Gaspar et al., 2001; Méthot et al., 2012; Rosati et al., 2012; Santini et al., 2018; Seebacher, 2001; Trites & Pauly, 1998). A complete set of references for the dataset is provided in Appendix S1. A standardized taxonomy, including unique species identifiers (AphiaID), synonymized names, and taxonomy was based on the World Register of Marine Species (WoRMS; WoRMS Editorial Board, 2023). In total, at least two estimates of maximum size were collected for 27,271 marine species from 24 phyla for a total of 69,570 size measurements. No quality control was conducted on size measurements. For example, if a species had a reported size well outside logical size range, we kept this error in the database because our objective was to specifically examine the influence of all errors, including typographical errors. As set out below, our results give some insight into the likely prevalence of such errors in large body size databases, and guidance for how to identify them. All data and code are available at https://anon.to/yrHI74.

For each species, the range among multiple measurements of maximum size was quantified as:

(1) $${\text{maxsize}}_{\text{range}}=\log_{10}\left({\text{maxsize}}_{\text{largest}}\right)-\log_{10}\left({\text{maxsize}}_{\text{smallest}}\right).$$

Note that this measure of range is the log‐transformed ratio of the maximum and minimum maximum size for each species. This calculation of range allows us to include in analyses species whose smallest and largest maximum sizes are equal (i.e., maxsize_range_ = 0).

Higher level taxonomy and broad habitat classifications (termed “functional groups” in WoRMS) for each species were taken from WoRMS (WoRMS Editorial Board, 2023). For the analyses, we combined habitat information into groups of benthic, pelagic, and unspecified/unknown (Table 1). WoRMS functional group data were compiled by expert taxonomic editors, with additional input from targeted pilot projects on specific taxa. Classification is at the species level, although can be at higher taxonomic levels for groups where all members are known to have the same broad functional group. Designations are typically unambiguous and in very few species is their disagreement between experts. For each species, we also coded whether it has an exoskeleton, endoskeleton, or no skeleton based on taxonomy and known invertebrate anatomy (Table 1). Count was taken as the number of maximum size estimates for each species.

**TABLE 1:** Summary of number of species per phylum, habitat group, and type of skeleton.

Phylum	Total count	Benthic	Pelagic	Unspecified	None	Exo	Endo
Annelida	379	372	1	6	379	0	0
Arthropoda	2601	594	1888	119	0	2601	0
Bryozoa	237	220	0	17	237	0	0
Chordata	13,080	9975	2927	178	0	0	13,080
Cnidaria	707	379	285	43	687	20	0
Echinodermata	234	228	0	6	0	234	0
Mollusca	9367	8600	235	532	763	8604	0
Nematoda	666	653	0	13	666	0	0
Totals	27,271	21,021	5336	914	2732	11,459	13,080

**FIGURE 1:**
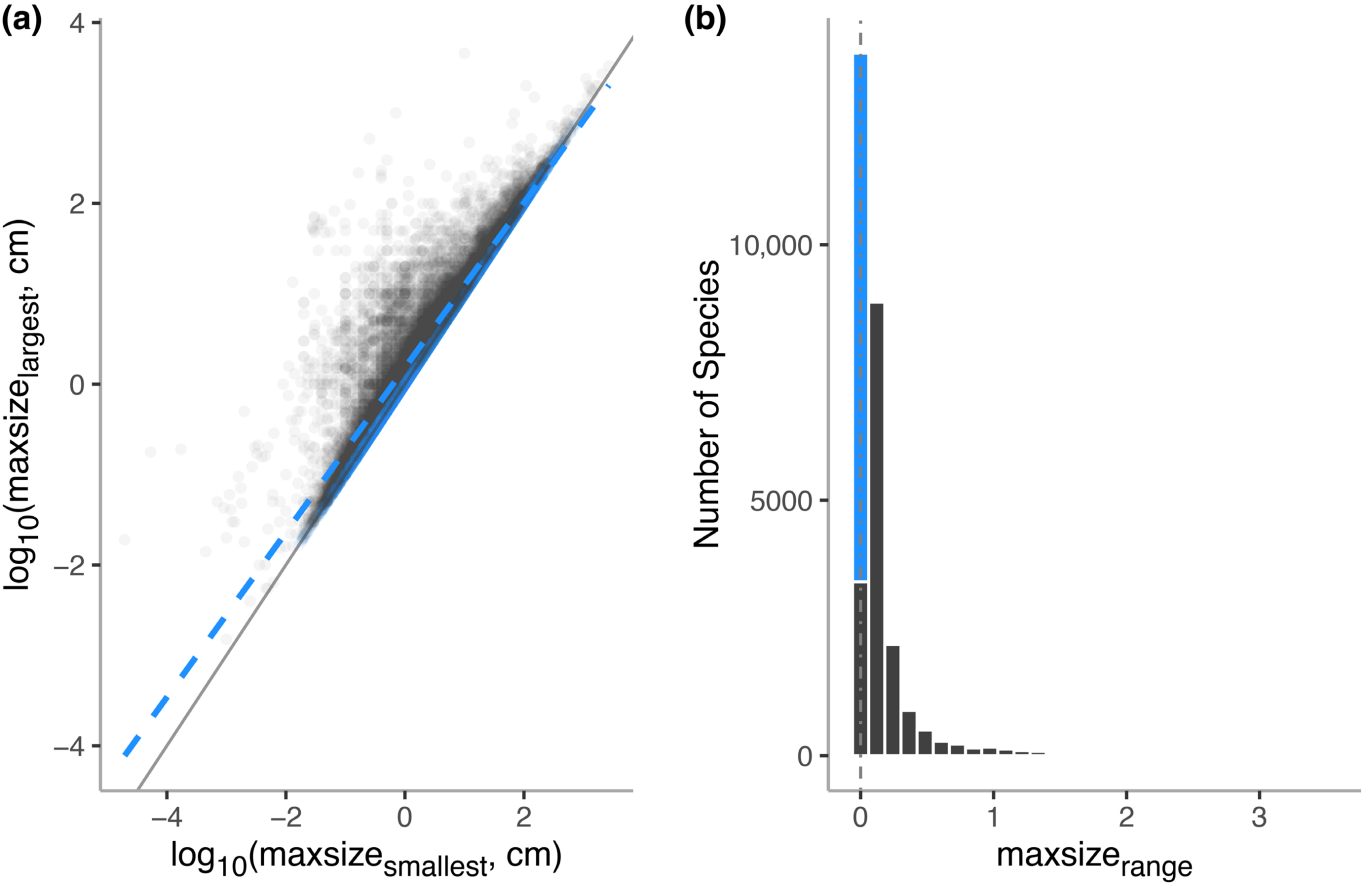
(a) Largest versus smallest reported maximum sizes for each individual species, that is, log_10_(maxsize_largest_, cm) versus log_10_(maxsize_smallest_, cm) for each individual species. Solid gray line represents 1:1 relationship between largest and smallest maximum reported size. Blue dash line is the linear model log_10_maxsize_largest_ = 0.17 + 0.91* log_10_maxsize_smallest_ between the two variables with zero uncertainty included, that is, log_10_maxsize_largest_ = log_10_maxsize_smallest_. (b) Distribution of size range, maxsize_range_, in length measurements (log_10_maxsize_largest_–log_10_maxsize_smallest_). Blue color represents species with no variation in measurements (maxsize_range_ = 0, *n* = 10,345 species).

For our main analyses, we limited the dataset to only include phyla with at least 100 species in our dataset to allow for robust statistical analysis (Table 1). This resulted in a final dataset of *n* = 27,271 species with a total of 69,570 measurements. The overall distribution of maxsize_range_ was heavily‐right skewed (most species have small ranges of maxsize_range_ but some have very large ranges) and also zero‐inflated (38% of species have zero variation in maxsize_range_ measures; Figure 1). Given this distribution of maxsize_range_, we analyzed the data using zero‐inflated gamma hurdle models, first using a binomial model with logit link to test which factors were associated with a species having zero or non‐zero size range, and then using a gamma model with log link to model the non‐zero values of size range (Brooks et al., 2017). We fitted these zero‐inflated gamma hurdle models using the function *glmmTMB* in the R package *glmmTMB* (Brooks et al., 2017), setting *family* to *ziGamma* with a log link, and using identical sets of predictors for both the zero and the non‐zero components of the model. Preliminary analyses showed that treating *count* as a continuous variable caused very high uncertainty in parameter estimates, especially at high values of *count*—which are rare in our dataset (the median value for *count* is 2, and only 755 (2.8%) species have >5 maximum size estimates). For all analyses, we therefore treated *count* as a four‐level categorical variable, with values 2, 3, 4–5, and ≥6.

Our models took the form:

(2) $$\begin{array}{c} {\text{maxsize}}_{\text{range}}\sim \log_{10}\left({\text{maxsize}}_{\text{smallest}}\right)+\text{count}+\text{variable}\\ +\log_{10}\left({\text{maxsize}}_{\text{smallest}}\right):\text{count}+\log_{10}\left({\text{maxsize}}_{\text{smallest}}\right):\text{variable} \end{array}$$

where variable is either phylum, habitat, invertebrate, or skeleton, or phylum and habitat, invertebrate and habitat, or skeleton and habitat. The categories were highly collinear (Table 1), particularly with phylum because the values of skeleton and invertebrate are largely conserved at high taxonomic scales. Because of this collinearity, we limited the set of predictors in any one model but ran all factors in different models. We compared separate models using sample‐corrected Aikake Information Criterion (AICc) values.

In all models, post hoc comparisons were conducted by computing estimated marginal means for specified factors or factor combinations in the general linear model and conducting contrasts among them using the function *eemeans* in the *eemeans* R package (Lenth, 2022). The function automatically adjusts for multiple comparisons using a Tukey adjustment.

To examine the impact of intraspecific variation in maximum size in broad‐scale comparative summaries of body size across all species, we compared the intraspecific variation in maximum size to variation observed at higher taxonomic levels, for the exemplar group of Gastropoda. We then considered the extent to which a species' position in the rank order of body sizes across all 27,271 species changes depending on which estimate of maximum size was chosen. We generated 1000 pairs of body size rankings, with each ranking obtained by drawing a single maximum size for each species. For each pair of rankings, we calculated the overall correlation in ranks, as well as the median and maximum changes in species rank position. We also identified the species with the largest change in body size ranking for each of the 1000 randomizations. Finally, we identified all species with a maxsize_range_ in excess of two orders of magnitude and investigated the reason for this large range.

## 3 RESULTS

A positive relationship, with a slope significantly less than one existed between maxsize_largest_ and maxsize_smallest_ for the full dataset of 27,581 species (maxsize_largest_ = 0.18 + 0.91* maxsize_smallest_, *t*‐ratio_(1,27,579)_ = −52.94, *p* < .0001, Adj. *R* ^2^ = .91 Figure 1a). After removing 10,345 species with no variation in measurements, the slope remained significantly less than one, with a slightly elevated intercept compared to the full dataset (maxsize_largest_ = 0.25 + 0.90* maxsize_smallest_, *t*‐ratio_(1,17,234)_ = −40.59, *p* < .0001, Adj. *R* ^2^ = .88 Figure 1a). Both the zero‐variation included and excluded datasets were significantly right‐skewed (D'Agostino skewness test, with zeros: skew = 4.76, *z* = 136.4, *p*‐value <.001; non‐zeros: skew = 4.03, *z* = 100.8, *p*‐value <.001, Figure 1b).

**FIGURE 2:**
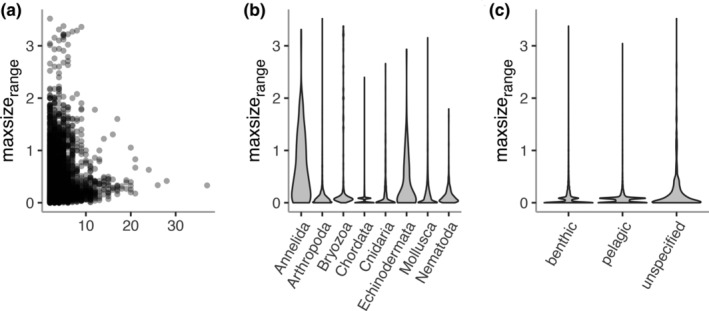
(a) Size range, maxsize_range_, in length measurements (log_10_maxsize_largest_–log_10_maxsize_smallest_) versus the number of measurements per species (b). Violin plots of maxsize_range_ by phylum. (c) Violin plots of maxsize_range_ by habitat.

Initial exploration of the eight phyla that have >100 species in our dataset suggested that range in maxsize_range_ varies with measurement count, phylum, and habitat (Figure 2). From the set of gamma hurdle models we fitted to test this, the model with the lowest AICc contained the predictors minimum size, count, phylum, and habitat, and the two‐way interactions between minimum size and each of the other variables (Table 2). This model substantially outperformed the second‐best model, which excluded *habitat* (Table 2). Models with skeleton performed worse than models with *phylum*.

**TABLE 2:** AICc for various Hurdle models to predict maxsize_range_. All Hurdle models also include maxsize_smallest_, count of observations, and interaction terms.

Model	df	AICc
Phylum + Habitat	53	−841.44
Phylum	45	486.00
Skeleton + Habitat	33	1684.91
Invertebrate + Habitat	29	2452.69
Habitat	25	3532.82
Skeleton	25	3816.72
Invertebrate	21	4591.70

**FIGURE 3:**
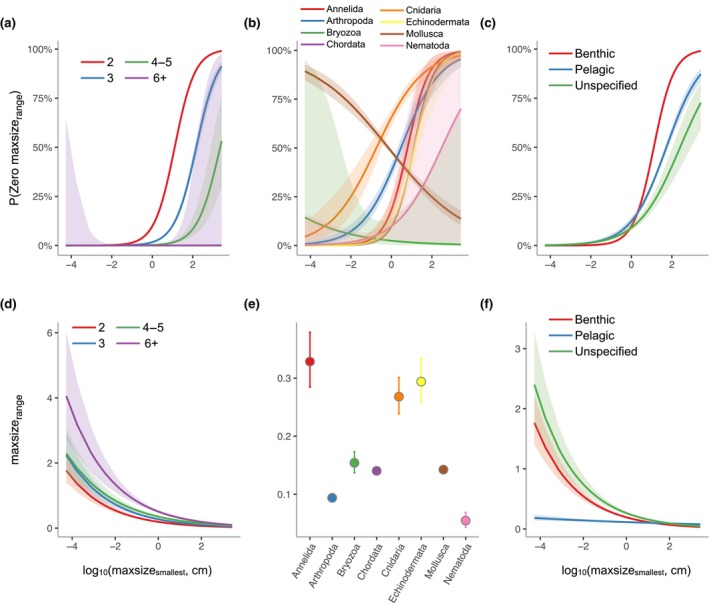
Predicted values (marginal effects) for specific model terms for the Hurdle model predicting range in maximum size estimates (maxsize_range_). (a) Effects of measurement count and log_10_maxsize_smallest_ on the probability of non‐zero maxsize_range_ in the zero‐inflation model (binary logistic regression) fit to all data. (b) Effects of phyla and log_10_maxsize_smallest_ on the probability of non‐zero maxsize_range_ in the zero‐inflation model (binary logistic regression) fit to all data. (c) Effects of habitat and log_10_maxsize_smallest_ on the probability of non‐zero maxsize_range_ in the zero‐inflation model (binary logistic regression) fit to all data. (d) Effects of measurement count and log_10_maxsize_smallest_) on maxsize_range_ in the conditional model (gamma regression model) fit to non‐zeros maxsize_range_ data. (e) Effects of phyla and log_10_maxsize_smallest_ on maxsize_range_ in the conditional model (gamma regression model) fit to non‐zeros maxsize_range_ data. (f) Effects of habitat and log_10_maxsize_smallest_ on maxsize_range_ in the conditional model (gamma regression model) fit to non‐zeros maxsize_range_ data.

The zero‐inflation component of the hurdle model (Table 3A) showed that the likelihood of a species having zero maxsize_range_ decreases with the number of maximum size estimates, but this relationship varied with maxsize_smallest_: the smallest species are very unlikely to have zero maxsize_range_ regardless of the value of count, whereas in larger species the likelihood of having zero maxsize_range_ approaches 100% for species with count = 2, declining to approximately 0% for species with high values of count (Figure 3a). In most phyla, the likelihood of having zero maxsize_range_ increases with increasing maxsize_smallest_ (Figure 3b), although note that the total range of sizes observed within most phyla spans only a part of the entire axis of maxsize_smallest_ (in particular, log_10_(maxsize_smallest_) is ≤0.38 for all nematodes). The exceptions to the general pattern are Mollusca and Bryozoa. For bryozoans, this exception is likely a consequence of small numbers of species with zero maxsize_range_, (2 species, or 0.8% of bryozoans). For mollusks however, the increased likelihood of zero maxsize_range_ in smaller species appears to be a genuine trend across the range of log_10_(maxsize_smallest_) observed in this group (c. −2 to +2.5). The relationship between the likelihood of zero maxsize_range_ and habitat also varied with maxsize_smallest_: at small sizes, zero maxsize_range_ is highly unlikely across all habitats, whereas at larger body sizes, zero maxsize_range_ is most likely for benthic species, followed by pelagic and then species with unspecified habitat (Figure 3c).

**TABLE 3:** Results of best Hurdle model to predict maxsize_range_. maxsize_range_ ~ maxsize_smallest_ + count + phylum + habitat + phylum* maxsize_smallest_ + habitat* maxsize_smallest_. Significant variables at the Bonferroni corrected value of *α* = .009 are in bold. Those variables significant at *α* = .05 are italicized. A. The zero‐inflation binomial model to test the association between each predictor and the likelihood of a species having zero variation in maximum size across all species. B. The conditional gamma model to test the association between each predictor and the value of maxsize_range_ across the species with non‐zero variation in maximum size.

A. Zero‐inflation model (Binary Logistic Regression All Data)	Estimate	Std. Error	*z* Value	Pr(>|*z*|)
**Intercept**	**−0.74**	**0.050**	**−14.8**	**<.0001**
**Maxsizesmallest**	**−0.61**	**0.064**	**−9.6**	**<.0001**
**Count3**	**0.34**	**0.019**	**18.2**	**<.0001**
**Count4‐5**	**0.60**	**0.021**	**29.1**	**<.0001**
**Count6+**	**0.97**	**0.034**	**28.2**	**<.0001**
**Arthropoda**	**−1.38**	**0.056**	**−24.7**	**<.0001**
**Bryozoa**	**−0.54**	**0.087**	**−6.1**	**<.0001**
**Chordata**	**−0.91**	**0.056**	**−16.1**	**<.0001**
**Cnidaria**	**−0.27**	**0.070**	**−3.9**	**.0001**
Echinodermata	0.10	0.097	1.0	.3003
**Mollusca**	**−0.92**	**0.051**	**−18.2**	**<.0001**
**Nematoda**	**−1.96**	**0.090**	**−21.8**	**<.0001**
**Pelagic**	**−0.53**	**0.024**	**−21.8**	**<.0001**
**Unspecified Habitat**	**0.31**	**0.037**	**8.4**	**<.0001**
Maxsizesmallest:Count3	0.03	0.019	1.3	.1848
**Maxsizesmallest:Count4‐5**	**0.08**	**0.024**	**3.4**	**.0006**
Maxsizesmallest:Count6+	0.03	0.041	0.8	.4406
**Maxsizesmallest:Arthropoda**	**0.21**	**0.069**	**3.1**	**.0022**
**Maxsizesmallest:Bryozoa**	**−0.36**	**0.078**	**−4.6**	**<.0001**
Maxsizesmallest:Chordata	0.10	0.067	1.4	.1546
Maxsizesmallest:Cnidaria	0.11	0.089	1.2	.2191
**Maxsizesmallest:Echinodermata**	**−0.34**	**0.124**	**−2.8**	**.0056**
Maxsizesmallest:Mollusca	0.14	0.066	2.1	.0361
**Maxsizesmallest:Nematoda**	**0.28**	**0.097**	**2.9**	**.0043**
**Maxsizesmallest:Pelagic**	**0.41**	**0.021**	**19.5**	**<.0001**
Maxsizesmallest:Unspecified Habitat	0.00	0.028	0.0	.9615

B. Conditional model (Gamma Regression Model to Non‐Zeros)	Estimate	Std. Error	*z* Value	Pr(>|*z*|)
**Intercept**	**−1.50**	**0.205**	**−7.29**	**<.0001**
**Maxsizesmallest**	**1.88**	**0.293**	**6.40**	**<.0001**
**Count3**	**−1.83**	**0.058**	**−31.5**	**<.0001**
**Count4‐5**	**−3.72**	**0.143**	**−26.0**	**<.0001**
**Count6+**	**−6.34**	**1.24**	**−5.09**	**<.0001**
**Arthropoda**	**1.01**	**0.221**	**4.58**	**<.0001**
**Bryozoa**	**−2.18**	**0.980**	**−2.23**	**.0260**
**Chordata**	**−0.72**	**0.216**	**−3.36**	**.0008**
**Cnidaria**	**2.14**	**0.225**	**9.51**	**<.0001**
Echinodermata	−0.60	0.441	−1.37	.1704
**Mollusca**	**1.42**	**0.207**	**6.88**	**<.0001**
Nematoda	−0.73	0.644	−1.13	.2582
**Pelagic**	**0.26**	**0.080**	**3.27**	**.0011**
Unspecified Habitat	−0.09	0.085	−1.04	.2969
**Maxsizesmallest:Count3**	**−0.14**	**0.052**	**−2.62**	**.0089**
**Maxsizesmallest:Count4‐5**	**−0.23**	**0.140**	**−1.66**	**.0979**
Maxsizesmallest:Count6+	−1.85	1.42	−1.30	.1937
**Maxsizesmallest:Arthropoda**	**−0.84**	**0.309**	**−2.73**	**.0063**
**Maxsizesmallest:Bryozoa**	**−2.32**	**0.749**	**−3.09**	**.0020**
Maxsizesmallest:Chordata	0.12	0.297	0.42	.6747
**Maxsizesmallest:Cnidaria**	**−1.01**	**0.318**	**−3.17**	**.0015**
Maxsizesmallest:Echinodermata	−0.01	0.472	−0.02	.9822
**Maxsizesmallest:Mollusca**	**−2.39**	**0.297**	**−8.06**	**<.0001**
Maxsizesmallest:Nematoda	−0.98	0.718	−1.37	.1718
**Maxsizesmallest:Pelagic**	**−0.87**	**0.061**	**−14.2**	**<.0001**
**Maxsizesmallest:Unspecified Habitat**	**−1.04**	**0.090**	**−11.6**	**<.0001**

The gamma model for non‐zero values of maxsize_range_ (Table 3B) showed that maxsize_range_ decreases with increasing maxsize_smallest_ and increases with increasing number of measurements (Figure 3d, Table S1). Values of maxsize_range_ were particularly high and variable in Annelida, Echinodermata, and Cnidaria, and lowest in Nematoda (Figure 3e, Appendix S2: Table S1). Values of maxsize_range_ were low in pelagic organisms regardless of size, and highest in small species with unspecified habitat, with benthic organisms intermediate (Figure 3f, Appendix S2: Table S2). These differences between habitats largely disappear among larger organisms (Figure 3f).

**FIGURE 4:**
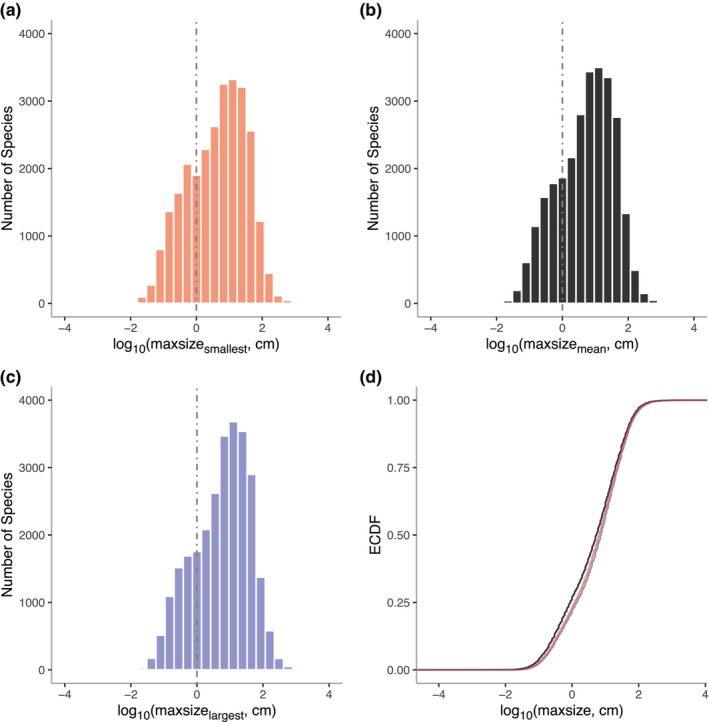
Distributions of reported sizes (a) log_10_maxsize_smallest_ (b) log_10_maxsize_mean_ (c) log_10_maxsize_largest_ for the complete dataset, (d) Empirical cumulative distribution function (ECDF) for log_10_maxsize_smallest_ (red), log_10_maxsize_mean_ (black), and log_10_maxsize_largest_ (blue).

**FIGURE 5:**
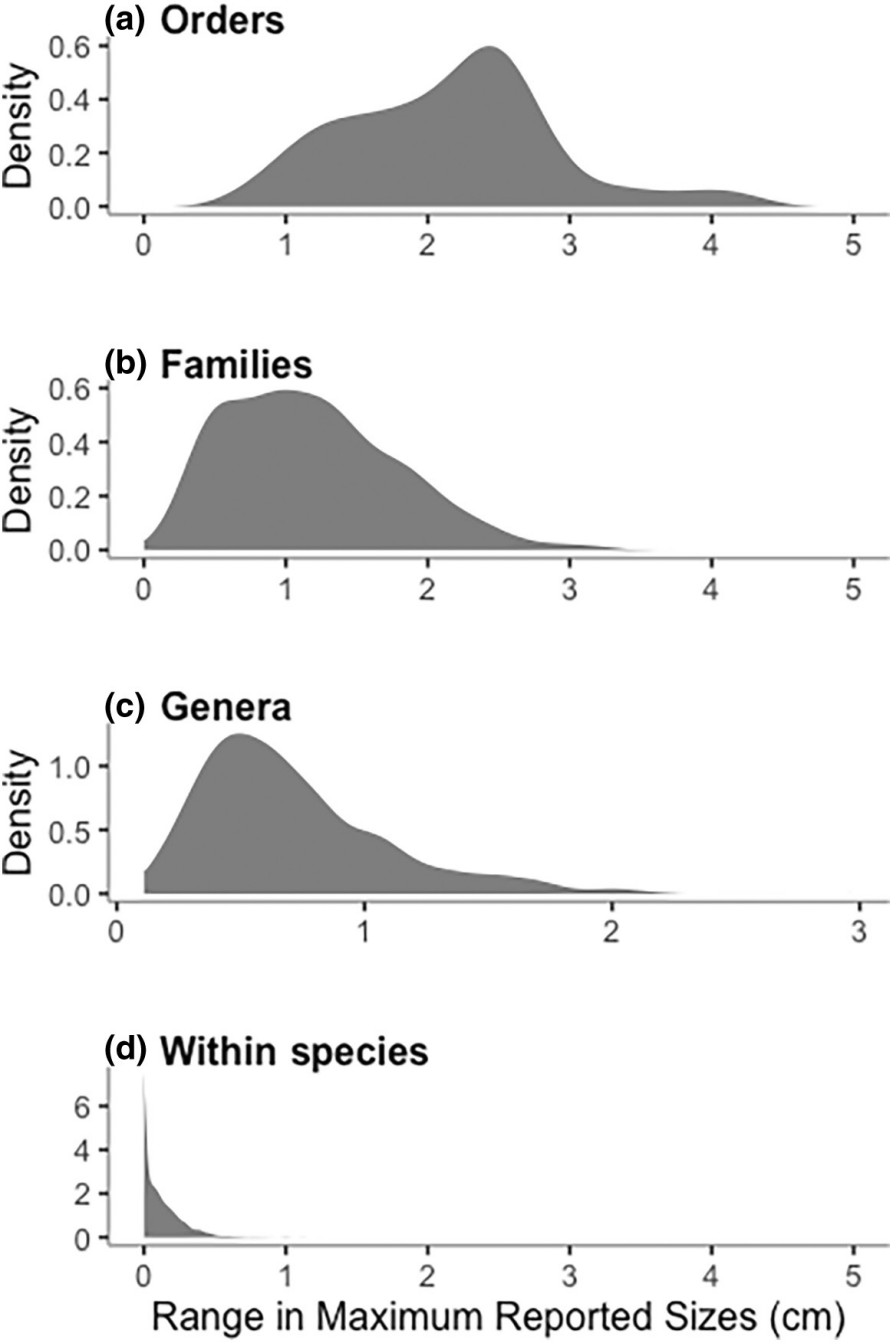
Distributions of size range, maxsize_range_, within gastropod (a) orders (*n* = 21) (b) families (*n* = 227) (c) genera (*n* = 944) (d) and species (*n* = 7059). Species size ranges reflect different reports of maximum size from different literature sources and databases. Size ranges for orders, families, and genera is the interspecific range between the largest and smallest species in the taxon.

Significant differences occurred between cumulative frequency distributions based on using maxsize_smallest_, maxsize_mean_, or maxsize_largest_ for a species (Figure 4, Table 4). Standard deviation of the maxsize distributions was the greatest in minimum and smallest in maximum measurements (Table 4). Distributions, once mean centered, also exhibited significant differences in variance (Table 4) except for between maxsize_largest_ and maxsize_mean_. However, visually the three distributions vary little from one another and Spearman's Rank Order Correlations are all highly significant, with rho >0.96 (Table 4 **).** Moreover, these variations in maxsize within a species are far less than the interspecific variation within genera, families, and orders, as exemplified for gastropods (Figure 5).

**TABLE 4:** Results of statistical tests between frequency distributions based minimum, mean, and maxsize.

Comparison	Mean (SD)	Kolmogorov–Smirnov Test (*D*, *p*‐value)	*F* test (on centered data) (*F*, *p*‐value)	Spearman's rank correlation (rho, *p*‐value)
Mean vs. Minimum	0.6846 (0. 8582)	0.0423, .001	1.0787, <.0001	.982, <.0001
	0.6010 (0.8948			
Mean vs. Maximum	0.6846 (0.8582)	0.0280, .001	1.0129, .2914	.998, <.0001
	0.7297 (0.8526)			
Minimum vs. Maximum	0.6010 (0.8948)	0.0621, .001	1.0926, < .0001	.967, <.0001
	0.7297 (0.8526)			

More generally, randomly drawing a single maximum size estimate for each species barely changes the overall rank order of species body sizes: the mean correlation between two such sets of rankings is 0.98 (*n* = 1000 randomizations), with the typical species changing body size rank due to intraspecific variation in maximum size only by around 24 places in the full rank order of all 27,571 species (Appendix S2: Figure S1). However, species with a very high maxsize_range_ can shift by 25,000 or more places in the body size rank order (Appendix S2: Table S3). We identified these species with very high maxsize_range_. Only 44 species (0.16%) varied in maximum size by over two orders of magnitude, 492 (1.8%) by one order of magnitude, and 1424 (5.2%) by half an order of magnitude. For the top 44 species (greater than two orders of magnitude in maxsize_range_), we further investigated the sources of variation (Appendix S2: Table S3). Bryozoa accounted for 19 of the 44 species with maxsize_range_ > 2, with a Bryozoan also the species with the biggest change in body size rank order in 916 of the 1000 randomizations described above. In these species, maxsize_largest_ represented a colony size and maxsize_smallest_ the dimension of an individual zooid. A similar issue arose with the six Bivalvia in this subset; all are members of wood‐boring families Xylophagiidae and Terenidae, where maxsize_smallest_ quantified the shell size and maxsize_largest_ the length of the foot. Unusually large maxsize_range_ occurred in two species of Echinodermata due to different measurements being the diameter of the central disc versus including arm length. This discrepancy was particularly noticeable in long‐armed Asteroidea. Five Polychaeta worms also had large maxsize_range_ due to both width and length measurements being reported as maxima. Three species of Chordata, all fishes, and five species of Cnidaria, all medusae or pelagic forms, had large maxsize_range_ that incorporated intraspecific size differences between adults or between adults and larvae. For example, *Praya dubia*, the giant siphonophore, has a maximum reported length of 45.72 m but also occurs in the database at 10 cm, a reasonable length for a small adult. *Pleuronectes platessa*, the flatfish European plaice, has a verifiable maximum size of 1.22 m but also occurs in the dataset as unlabeled juvenile fish of 1.1 cm. The remaining four species of the 44 represent true errors in size including two species Polychaeta, a species of Chordata, and one species of Copepoda. For example, one of the Polychaeta, *Polyophthalmus mauliola*, is a small worm from subtidal mudflats with the holotype measuring 7.5 mm (MagalhÃes et al., 2019) but occurs in the dataset here as 51.83 m long. The Chordata species, Fraser's dolphin, *Lagenodelphis hosei* has a maxsize_smallest_ of 2.6 cm. In both cases, the error is most likely to result from incorrect specification of measurement units.

## 4 DISCUSSION

Here, we analyzed reported maximum size measurements of 27,271 marine species and aimed to understand the factors influencing variation in these estimates. Range in maximum size within a species scaled from zero, that is, multiple sources gave identical measurements for maximum size, to over three orders of magnitude. The results highlight the complexity and potential sources of variability in body size measurements emphasizing the influence of: (1) organism size, (2) taxonomic group, (3) habitat, and (4) measurement methodology on the reported values of maximum linear dimension within marine species.

### 4.1 Organism size

The relationship between the smallest and largest maximum size per species had an observed slope less than one (*b* = 0.91), indicating greater variation associated with size measurements in smaller species, which may be attributed to several factors. First, measurement error becomes more pronounced as it approaches the scale of measurement, leading to increased variability in size estimates. From our experience with extracting maximum size data, sizes of smaller organisms are often rounded off or not measured to a level of precision corresponding with the size of the organism. However, this phenomenon of increased uncertainty due to rounding appears to be limited to organisms below 100 micrometers (equivalent to a log_10_ size of −2 in Figure 3a). Another possibility is that smaller organisms may have less mature taxonomy, implying a less refined categorization compared to vertebrates and macroinvertebrates. This potential disparity in taxonomic knowledge could arise from the relatively younger field and involvement of fewer researchers. Certainly, the most recently described marine species tend to be relatively small, with the modal size class of marine species described between 2013 and 2017 at 2–10 mm (Bouchet et al., 2023). Bouchet et al. (2023) also document high rates of synonymy across marine species, up to 25% for species described between 1910 and 1950. If these rates of synonymy were biased toward smaller species, this phenomenon could lead to a greater maxsize_range_ once aggregated to the newly synonymized species level. Regardless, taxonomic uncertainty and revision can lead to substantial variation in maxsize: eight currently accepted marine species are listed in Hayward and Ryland (1990) under two or more synonyms with separate estimates of maxsize, with this issue of synonymy alone resulting in maxsize_range_ values of up to 0.56, a *c*. 3.6× difference between smallest and largest maximum size (TJW unpublished analyses).

### 4.2 Taxonomic group

Echinoderms, annelids, and bryozoans exhibited significantly greater variation in maxsize_range_ when compared to other phyla (Figure 3 **)**. Notably, mollusks, chordates, arthropods, and nematodes displayed the smallest differences in maxsize_range_. The variation in measures of maxsize within echinoderms might be attributed to the way size is recorded. For instance, Hayward and Ryland (1990) list maxsize for the classes within Echinodermata as variously “arm length” (Crinoidea), “diameter” (Asteroidea), “disc diameter” (Ophiuroidea) “test diameter” (Echinoidea), and “total length” (Holothurioidea). Similarly, it is plausible that the same consideration regarding measurement techniques applies to cnidarians, and perhaps certain annelids such as the feather‐duster (family Sabellidae). Another factor potentially influencing the observed maxsize_ranges_ is the preservation of small organisms within these phyla. Preservation methods may have a differential impact on size estimation, leading to variations in the recorded size range. Importantly, the influence of phylum on observed size ranges is larger than the influences of other contributing factors. Further investigation and understanding of these differences can shed light on the underlying mechanisms shaping size variations within and among phyla.

### 4.3 Habitat type

The analysis of maxsize_range_ across different habitat types revealed notable distinctions. Interestingly, benthic organisms displayed a relatively higher level of maxsize_range_ compared to other habitat types. It is possible that this discrepancy arises from the challenge of distinguishing measurement errors from ecophenotypic variations, given the inherent variability of benthic habitats. These habitats may exhibit greater habitat diversity compared to pelagic or demersal environments. Benthic species dominate marine biodiversity and occur in more animal phyla than other functional groups (Webb & Vanhoorne, 2020). The range of body forms and morphologies this encompasses likely exacerbates some of the methodological issues previously raised. For instance, almost all Echinodermata in our dataset (228 of 234 species) are benthic, and the use of different length measurements by different researchers may increase variation within this group. Species with unspecified habitats exhibited the highest maxsize_range_; the lack of comprehensive ecological data suggests these organisms have been poorly explored, likely leading to less‐well‐constrained size measurements.

### 4.4 Measurement methodology

Increasing the number of measurements decreases the likelihood of zero variation, and once non‐zero variation exists, a further increase in measurements can lead to an increase in overall variation. While this observation may present challenges, it also highlights the need to carefully consider measurement strategies and their impact on size estimates. The persistence of this issue rests on the absence of a centralized repository for size data, resulting in multiple measurements scattered across separate databases and publications. To address this dispersion of information, the creation of a curated, centralized, industry‐standard database for size data among marine species would prove highly beneficial, enabling the reconciliation of various maximum size measures and facilitating error detection and correction processes.

### 4.5 Can we use maximum size in macro‐ecological and ‐evolutionary research?

An analysis of cumulative frequency distributions based on maxsize_smallest_, maxsize_largest_, maxsize_range_ estimates for each species revealed significant differences among these distributions. Specifically, the maxsize values exhibited a consistent increase in discrepancy from the smallest to the mean estimates, and further to the largest estimates. Additionally, the distributions displayed variation in terms of their variance. The distinction between the largest and the smallest reported maximum size as measures of size range deserves consideration. Although the use of maximum size has been criticized in interspecific comparative analyses, it remains a common practice due to limitations in available data and the assumption that among‐species differences outweigh within‐species differences. This study provides empirical evidence that choice of measurement can change the nature of the distribution, that is, picking the largest known measurement for each species may significantly shift the distribution.

However, we also show that these differences in size distributions, while significant, are minor and subtle. We also demonstrate that these differences in maximum size estimates also do not significantly change the rank order sizes of species, which would allow for robust eco‐evolutionary examination. The variation in maximum size estimates is also far less than the natural variation in maximum size within even small, low‐diversity clades, and the data are suitable for evaluating changes in mean with large sample sizes, particularly in changes larger than one log unit or more. For any particular case, our data provide guidance to assess whether the signal being assessed is likely to exceed the noise inherent in using maximum size values. We do identify some species with very large ranges in maximum size (Appendix S2: Table S3), however these are unusual cases and do not preclude the use of large compilations of species‐level maximum size in comparative macroecology and macroevolution.

## 5 CONCLUSIONS

Our results indicate that actual errors in estimates of maximum body size reported in the literature are rare (<2%) and many inconsistences in maximum size can be accounted for with better annotation. This variation in reports of maximum size within species is also far less than interspecific variation and in most macroecological and macroevolutionary studies it is unlikely to impact the results. However, it is essential to address the practical utility of current data and identify the specific research questions and effect sizes for which the available variation and uncertainty can still provide meaningful insights. Clarifying these aspects will contribute to a more nuanced understanding of the signal‐to‐noise issue in body size datasets and foster better‐informed interpretations of study outcomes. Further, we reiterate that establishing standardized measurement protocols and promoting the sharing of size data through a curated centralized repository, would represent a substantial advantage for the research community.

## AUTHOR CONTRIBUTIONS

**Craig R. McClain:** Conceptualization (lead); data curation (lead); investigation (lead); methodology (lead); project administration (lead); validation (lead); visualization (lead); writing – original draft (lead); writing – review and editing (lead). **Thomas J. Webb:** Data curation (supporting); formal analysis (supporting); investigation (supporting); methodology (supporting); validation (supporting); visualization (supporting); writing – original draft (supporting); writing – review and editing (supporting). **Noel A. Heim:** Data curation (supporting); formal analysis (supporting); methodology (supporting); validation (supporting); writing – original draft (supporting); writing – review and editing (supporting). **Matthew L. Knope:** Conceptualization (supporting); methodology (supporting); writing – original draft (supporting); writing – review and editing (supporting). **Pedro M. Monarrez:** Conceptualization (supporting); data curation (supporting); methodology (supporting); writing – original draft (supporting); writing – review and editing (supporting). **Jonathan L. Payne:** Conceptualization (supporting); data curation (supporting); methodology (supporting); writing – original draft (supporting); writing – review and editing (supporting).

## FUNDING INFORMATION

None.

## CONFLICT OF INTEREST STATEMENT

None Declared.

## Supporting information


Appendix S1.

Appendix S2.

## Data Availability

Data are available at https://github.com/crmcclain/MOBS_OPEN/.
