# Supplementary material for: Navigating uncertainty in maximum body size in marine metazoans
Source: Ecol Evol. 2024 Jun 5;14(6):e11506. doi: 10.1002/ece3.11506 (PMC11151150; doi:10.1002/ece3.11506)
Supplement: Supplementary file 1 — Appendix S1. [file ECE3-14-e11506-s001.docx]

| Citation | Number of Measurements |
| --- | --- |
| (Ahyong et al., 2023) | 29,381 |
| (Froese & Pauly, 2022) | 12,431 |
| (Palomares & Paulay, 2022) | 7,470 |
| (Liggia, 2013) | 5,888 |
| (McClain, Gullett, Jackson‐Ricketts, & Unmack, 2012) | 4,063 |
| (Turton, 1932) | 1,348 |
| (Keen, 1971) | 1,073 |
| (Encylopedia of Life, 2022) | 941 |
| (Kramp, 1961) | 655 |
| (Liow & Taylor, 2019) | 648 |
| (Roper, Sweeney, & Nauen, 1984) | 516 |
| (Kay, 1979) | 488 |
| (Balk & McClain, 2022) | 482 |
| (Fisher, 1952) | 343 |
| (Merle, Garrigues, & Pointier, 2011) | 291 |
| (Peñas & Rolán, 2017) | 257 |
| (Yidi Daccarett & Bossio, 2011) | 239 |
| (J. Bailey-Brock & Gerstle, 2023) | 193 |
| (W. J. Clench, 1946-1953) | 136 |
| (W. J. Clench, 1960-1970) | 117 |
| (Bartsch, 1912) | 116 |
| (C. A. Allgén, 1959) | 92 |
| (C. A. Allgén, 1957) | 90 |
| (Wilson, 1994) | 83 |
| (H. Platt, Warwick, & Furstenberg, 1985) | 80 |
| (Gracia, Adrila, & Diaz, 2004) | 75 |
| (J. J. v. Aartsen, Gittenberger, & Goud, 1998a) | 67 |
| (Bartsch, 1917) | 64 |
| (Taylor & Walls, 1975) | 62 |
| (Bartsch, 1927) | 52 |
| (J. J. V. Aartsen, Gittenberger, & Goud, 1998b) | 52 |
| (Spurgeon, 2021) | 50 |
| (Iredale, 1936) | 49 |
| (Hoffman, Gofas, & Freiwald, 2020) | 45 |
| (Baker, Hanna, & Strong, 1928) | 42 |
| (Verhecken, 2011) | 41 |
| (Bartsch, 1926) | 40 |
| (Stekhoven, 1950) | 38 |
| (R. C. Moore & Pitrat, 1961) | 37 |
| (Dall & Bartsch, 1909) | 34 |
| (Ellis & Messina, 1952); (Ellis & Messina, 1940) | 33 |
| (Bartsch, 1915) | 32 |
| (Bartsch, 1928) | 32 |
| (Houbrick, 1992) | 32 |
| (Fahey & Gosliner, 2004) | 26 |
| (Høisæter, 2014) | 26 |
| (Kantor, Harasewych, & Puillandre, 2016) | 26 |
| (Filip, 1969) | 25 |
| (Thiele, 1925) | 24 |
| (Carmona, Bhave, et al., 2014) | 23 |
| (Valdes, 2002) | 23 |
| (Winston F Ponder & Worsfold, 1994) | 23 |
| (Carmona, Pola, Gosliner, & Cervera, 2014b) | 22 |
| (Węsławski, Legeżyńska, & Włodarska‐Kowalczuk, 2020) | 22 |
| (Terrence M. Gosliner & Johnson, 1994) | 20 |
| (Bartsch, 1920) | 19 |
| (Kano, Chikyu, & Warén, 2009) | 19 |
| (P. Bartsch, 1911) | 18 |
| (Timm, 1952) | 18 |
| (Wolfgang Wieser, 1954) | 18 |
| (Nomura, 1936) | 17 |
| (Tuskes & Tuskes, 2019) | 17 |
| (Weiser, 1956) | 17 |
| ("Sea Slug Forum," 2010) | 16 |
| (Schuurmans Stekhoven, Adam, & De Coninck, 1931) | 16 |
| (Tuskes, 2019) | 16 |
| (Venkataraman, 2013) | 16 |
| (W Wieser, 1953) | 16 |
| (Beechey, 2023) | 15 |
| (Dall & Bartsch, 1906) | 15 |
| (Peñas, Rolán, & Swinnen, 2014) | 15 |
| (Arthur William Baden Powell, 1937) | 14 |
| (Bartsch, 1922) | 13 |
| (Daniel C Cavallari, Salvador, Dornellas, & Simone, 2019) | 13 |
| (John Hemsworth Day, 1967) | 13 |
| (Mello & Maestrati, 1986) | 13 |
| (Anders Warén, 1992) | 12 |
| (Pizzini, Raines, & Vannozzi, 2013) | 12 |
| (Absalão & dos Santos Gomes, 2001) | 11 |
| (B. Marshall, 1979) | 11 |
| (Guido T. Poppe, Tagaro, & Stahlschmidt, 2015) | 11 |
| (J. Ortea, Moro, & Espinosa, 2015) | 11 |
| (T. Gosliner, 2015) | 11 |
| (Turner, 2002) | 11 |
| (Barnard, 1963) | 10 |
| (Charles Francis Laseron, 1951) | 10 |
| (Dornellas, 2012) | 10 |
| (Issel, 1869) | 10 |
| (J Ortea, Moro, Caballer, & Hernández, 2003) | 10 |
| (Dell, 1956) | 9 |
| (Gerlach, 1953) | 9 |
| (Gofas, Luque, Oliver, Templado, & Serrano, 2021) | 9 |
| (Goodey, 1963) | 9 |
| (Marincovich, 1973) | 9 |
| (Carmona, Pola, Gosliner, & Cervera, 2017) | 8 |
| (Daniel Caracanhas Cavallari, Almeida, & Simone, 2020) | 8 |
| (García-Gómez, Cimino, & Medina, 1990) | 8 |
| (Poulin, 1995) | 8 |
| (W. Clench & Turner, 1946) | 8 |
| (B. K. Raines, 2020) | 7 |
| (C. F. Laseron, 1959) | 7 |
| (Ch F Laseron, 1954) | 7 |
| (Claude Vilvens, 2005) | 7 |
| (D. R. Moore, 1972) | 7 |
| (Kilburn, 1973) | 7 |
| (Miller, 2001) | 7 |
| (Saurin, 1959) | 7 |
| (A Warén, 1993) | 6 |
| (Angas, 1878) | 6 |
| (B. Marshall, 1988) | 6 |
| (Baba, 1976) | 6 |
| (Bidgrain, 2020) | 6 |
| (Brazier, 1877) | 6 |
| (C. Allgén, 1954) | 6 |
| (Cheeseman, 1881) | 6 |
| (Cooke et al., 2014) | 6 |
| (Dautzenberg, I, Fischer, & de Boury, 1896) | 6 |
| (H. A. Pilsbry & McGinty, 1949) | 6 |
| (Rubio & Rolán, 2013) | 6 |
| (Yonow & Jensen, 2018) | 6 |
| (B. A. Marshall, 1988) | 5 |
| (Burn, 1969) | 5 |
| (Carmona, Lei, et al., 2014) | 5 |
| (Carmona, Pola, Gosliner, & Cervera, 2014a) | 5 |
| (Charles F Laseron, 1958) | 5 |
| (G.T. Poppe & Poppe, 2023) | 5 |
| (H. M. Platt, 1982) | 5 |
| (Habe, 1976) | 5 |
| (J Ortea, Moro, & Bacallado, 2006) | 5 |
| (Locard, 1897) | 5 |
| (MagalhÃes et al., 2018) | 5 |
| (McLean & Andrade, 1982) | 5 |
| (P. Bouchet, 1991) | 5 |
| (Peñas & Rolán, 1997) | 5 |
| (Philippe Bouchet & Kilburn, 1991) | 5 |
| (Salvador & Cunha, 2016) | 5 |
| (T. Gosliner, 2011) | 5 |
| (W. Ponder, 1965a) | 5 |
| (Waren & Bouchet, 1993) | 5 |
| (A. W. B. Powell, 1940) | 4 |
| (Allan, 1933) | 4 |
| (Anders Warén, 1996) | 4 |
| (Caballer, Ortea, & Redfern, 2014) | 4 |
| (Claude Vilvens, 2014) | 4 |
| (Dall & Bartsch, 1904) | 4 |
| (Dall, 1913) | 4 |
| (Dall, 1919) | 4 |
| (G. Sowerby, 1894) | 4 |
| (H. A. Pilsbry, 1917) | 4 |
| (Houbrick, 1980) | 4 |
| (Jesús Ortea & Moro, 2018) | 4 |
| (Kienberger et al., 2016) | 4 |
| (MagalhÃes, Bailey-Brock, & Watling, 2018) | 4 |
| (Moro & Ortea, 2015) | 4 |
| (Pizzini & Raines, 2011) | 4 |
| (Tenison Woods, 1875) | 4 |
| (Van der Linden & Wagner, 1990) | 4 |
| (Watson, 1879) | 4 |
| (Absalao, Miyaji, & Pimenta, 2001) | 3 |
| (Albano & Pizzini, 2011) | 3 |
| (B. Raines & Pizzini, 2005) | 3 |
| (Boughet & Warén, 1979) | 3 |
| (Brown, 1827) | 3 |
| (C Hedley, 1907) | 3 |
| (C Vilvens, 2012) | 3 |
| (Charles Hedley, 1912) | 3 |
| (Connolly, 1939) | 3 |
| (Crane, 1975) | 3 |
| (E. A. Smith, 1904) | 3 |
| (E. Smith, 1907) | 3 |
| (Eliot, 1903) | 3 |
| (Filipʹev, 1973) | 3 |
| (Güller & Zelaya, 2019) | 3 |
| (Habe, 1952) | 3 |
| (Iredale, 1912) | 3 |
| (James Cosmo Melvill, 1910) | 3 |
| (Jeffreys, 1883) | 3 |
| (Jesús Ortea, Moro, Bacallado, & Caballer, 2014) | 3 |
| (Okutani & Fujikura, 2002) | 3 |
| (Padula & Delgado, 2010) | 3 |
| (Paramonov, 1976) | 3 |
| (Perrone, 1990) | 3 |
| (Philippe Bouchet, 1975) | 3 |
| (Pimenta, Santos, & AbsalÃO, 2011) | 3 |
| (Pittman & Fiene, 2023) | 3 |
| (R. Bergh, 1905) | 3 |
| (R. Timm, 1976) | 3 |
| (Reeve, 1842) | 3 |
| (Rolán & Rubio, 2002) | 3 |
| (Saurin, 1958) | 3 |
| (Saurin, 1961) | 3 |
| (Tanamai & Nabhitabhata, 2016) | 3 |
| (Tate & May, 1900) | 3 |
| (Thiele, 1912) | 3 |
| (Vannozzi, Pizzini, & Raines, 2015) | 3 |
| (William Healey Dall, 1908) | 3 |
| (Brazier, 1894) | 2 |
| (Breslau, Valdés, & Chichvarkhin, 2016) | 2 |
| (Corgan, 1972) | 2 |
| (Cunha & Simone, 2018) | 2 |
| (Di Geronimo, Privitera, & Valdovinos, 1995) | 2 |
| (Edmunds, 2015) | 2 |
| (Giere, Ebbe, & Erséus, 2008) | 2 |
| (H. Pilsbry, 1949) | 2 |
| (Hertlein & Strong, 1951) | 2 |
| (Hori & Fukuda, 1999) | 2 |
| (Hornung & Mermod, 1924) | 2 |
| (Hutton, 1885) | 2 |
| (J. Bailey-Brock & Hartman, 1987) | 2 |
| (J. H. Bailey-Brock, 1991) | 2 |
| (J. H. Bailey-Brock, Dreyer, & Brock, 2003) | 2 |
| (J. v. Aartsen & Bogi, 1988) | 2 |
| (Jesus Ortea & Moro, 2009) | 2 |
| (Jesus Ortea, Luque, & Templado, 1990) | 2 |
| (Killeen & Oliver, 2000) | 2 |
| (Knudsen, 1964) | 2 |
| (Korshunova, Martynov, et al., 2017) | 2 |
| (Korshunova, Zimina, & Martynov, 2017) | 2 |
| (Krug, Morley, Asif, Hellyar, & Blom, 2008) | 2 |
| (Linden & Eikenboom, 1992) | 2 |
| (Magalhães, Bailey-Brock, & Santos, 2015) | 2 |
| (Mehrotra et al., 2021) | 2 |
| (Mimoto & Nakao, 2009) | 2 |
| (Mörch, 1863) | 2 |
| (Murina, 1968) | 2 |
| (O’Donoghue, 1929) | 2 |
| (P Bouchet, 1977) | 2 |
| (Pelorce, Horst, & Hoarau, 2013) | 2 |
| (Peñas & Rolán, 1999) | 2 |
| (Perkins, 1981) | 2 |
| (Pilsbury, 1893-1895) | 2 |
| (Rehder & Ladd, 1973) | 2 |
| (Renda & Micali, 2016) | 2 |
| (Risbec, 1928) | 2 |
| (Rudman, 1971) | 2 |
| (Schander, 1994) | 2 |
| (T. M. Gosliner, 1985) | 2 |
| (Tennison-Woods, 1874) | 2 |
| (Terrence M Gosliner, 1990) | 2 |
| (Terrence M Gosliner, 2010) | 2 |
| (Test, 1945) | 2 |
| (Verco, 1904) | 2 |
| (W H Dall, 1908) | 2 |
| (W. Ponder, 1965b) | 2 |
| (Waren, 1989) | 2 |
| (White, 1946) | 2 |
| (Yokoyama, 1922) | 2 |
| (Yonow, 1994) | 2 |
| (A. A. Gould, 1852) | 1 |
| (A. A. Gould, 1861) | 1 |
| (Audouin, 1832) | 1 |
| (Augener, 1922) | 1 |
| (Augustus Addison Gould, 1849) | 1 |
| (B. A. Marshall, 1996) | 1 |
| (B. K. Raines, 2019) | 1 |
| (Bartsch, 1923) | 1 |
| (Bebbington, 1974) | 1 |
| (Blake, 1979) | 1 |
| (Bruce A Marshall & Walton, 2019) | 1 |
| (Burn, 2015) | 1 |
| (Carpenter, 1866) | 1 |
| (CHARLES Hedley, 1906) | 1 |
| (Chichvarkhin, 2016) | 1 |
| (Clarke Jr & Menzies, 1959) | 1 |
| (Daniel C Cavallari, Salvador, & Simone, 2014) | 1 |
| (de La Saussaye, 1869) | 1 |
| (Deel, 2000) | 1 |
| (E. A. Smith, 1910) | 1 |
| (E. Smith, 1904) | 1 |
| (Espinosa & Ortea, 2011) | 1 |
| (Fauchald, Granados-Barba, & Solís-Weiss, 2009) | 1 |
| (G. B. Sowerby, 1892) | 1 |
| (G. B. Sowerby, 1897) | 1 |
| (G. Sowerby, 1900) | 1 |
| (Garrett, 1873) | 1 |
| (Gofas & Hoffman, 2020) | 1 |
| (H. Pilsbry & McGinty, 1950) | 1 |
| (H. Pilsbury, 1917) | 1 |
| (Habe, 1958a) | 1 |
| (Habe, 1958b) | 1 |
| (Hartman, 1960) | 1 |
| (Hartmann-Schröder, 1979) | 1 |
| (Hertz, 1994) | 1 |
| (Hylleberg, 2013) | 1 |
| (I. Bartsch, 1911) | 1 |
| (Imajima, 1972) | 1 |
| (J Ortea et al., 2006) | 1 |
| (J Ortea, Moro, & Espinosa, 1996) | 1 |
| (J. Cosmo Melvill, 1896) | 1 |
| (J. Cosmo Melvill, 1906) | 1 |
| (J. H. Bailey-Brock, Jouin-Toulmond, & Brock, 2010) | 1 |
| (J. J. V. Aartsen & Smith, 1996) | 1 |
| (Jeffreys, 1860) | 1 |
| (Jesús Ortea, Valdés, & García-Gómez, 1996) | 1 |
| (John H Day, 1961) | 1 |
| (Kano et al., 2009) | 1 |
| (Kantor, Kosyan, Sorokin, Herbert, & Fedosov, 2020) | 1 |
| (Kirkegaard, 1959) | 1 |
| (L. S. R. Bergh, 1878) | 1 |
| (Ladd, 1966) | 1 |
| (Langerhans, 1881) | 1 |
| (Laws, 1937) | 1 |
| (Laws, 1941) | 1 |
| (Lima, Simone, & Guimarães, 2016) | 1 |
| (Magalhaes, Bailey-Brock, & Rizzo, 2012) | 1 |
| (Magalhaes, Bailey–Brock, & Davenport, 2011) | 1 |
| (Miller, 1987) | 1 |
| (Oug, 1978) | 1 |
| (Pastorino & Chiesa, 2014) | 1 |
| (Pease, 1871) | 1 |
| (Peñas & Rolán, 1998) | 1 |
| (Peñas & Rolán, 2002) | 1 |
| (Pettibone, 1963) | 1 |
| (Pizzini, Nofroni, & Bonfitto, 2008) | 1 |
| (R. W. Timm, 1976) | 1 |
| (Rato, Gutiérrez, Abad, & Espinosa, 2014) | 1 |
| (Raveendran & Wagh, 1991) | 1 |
| (San Martín, 2005) | 1 |
| (Sasaki, 2008) | 1 |
| (Schepman, 1913) | 1 |
| (Smriglio & Mariottini, 1996) | 1 |
| (Souverbie & Montrouzier, 1865) | 1 |
| (Strong & Bouchet, 2013) | 1 |
| (Suter, 1898) | 1 |
| (Treadwell, 1906) | 1 |
| (Treadwell, 1941) | 1 |
| (ValdÉs, Lundsten, & Wilson, 2018) | 1 |
| (Van Aartsen, 2008) | 1 |
| (Vannozzi, 2016) | 1 |
| (Vélain, 1887) | 1 |
| (Verco, 1909) | 1 |
| (W. Clapp, 1924) | 1 |
| (W. F. Clapp, 1923) | 1 |
| (W. F. Ponder, 1999) | 1 |
| (W. Ponder & Yoo, 1976) | 1 |
| (W. Ponder, 1968) | 1 |
| (Zelaya, Schejter, & Ituarte, 2011) | 1 |

Literature Cited

1. Aartsen, J. J. v., Gittenberger, E., & Goud, J. (1998a). Pyramidellidae (Mollusca, Gastropoda, Heterobranchia) collected during the Dutch CANCAP and MAURITANIA expeditions in the south-eastern part of the North Atlantic Ocean (part 1). CANCAP-project. Contributions, no. 119. *321*(1), 1-57.
2. Aartsen, J. J. V., Gittenberger, E., & Goud, J. (1998b). Pyramidellidae (Mollusca, Gastropoda, Heterobranchia) collected during the Dutch CANCAP and MAURITANIA expeditions in the south-eastern part of the North Atlantic Ocean (part 2). *74*, 1-50.
3. Aartsen, J. J. V., & Smith, S. M. (1996). Odostomia harveyi spec. nov. from the Northern Atlantic Ocean (Gastropoda, Pyramidellidae). *60*(4/6), 149-151.
4. Aartsen, J. v., & Bogi, C. (1988). Anekes gittenbergeri and Anekes nofronii, two new gastropods from the Mediterranean. *Bolletino Malacologico, 24*, 27-32.
5. Absalão, R. S., & dos Santos Gomes, R. (2001). The species usually reported in the subgenus *Brochina* (Caecum, Caecidae, Caenogastropoda) from Brazil and some relevant type specimens from western Atlantic. *Bollettino Malacologico, 37*(1/4), 9-22.
6. Absalao, R. S., Miyaji, C., & Pimenta, A. D. (2001). The genus *Brookula* Iredale, 1912 (Gastropoda, Trochidae) from Brazil: description of a new species, with notes on other South American species. *ZOOSYSTEMA-PARIS-, 23*(4), 675-688.
7. Ahyong, S., Boyko, C. B., Bailly, N., Bernot, J., Bieler, R., Brandão, S. N., . . . Zullini, A. (2023). World Register of Marine Species (WoRMS). Retrieved 2023-03-23, from WoRMS Editorial Board https://www.marinespecies.org
8. Albano, P. G., & Pizzini, M. (2011). Notes on Caecidae (Mollusca: Gastropoda) from southern Mozambique, with the description of a new species. *African Invertebrates, 52*(1), 1-10.
9. Allan, J. K. (1933). Opisthobranchs from Australia. *Records of the Australian Museum, 18*(9), 443-450.
10. Allgén, C. (1954). Freeliving marine nematodes from East Greenland and Jan Mayen. The Swedish Greenland Expedition 1899. *Meddelelser om Gronland, 107*(6), 1-44.
11. Allgén, C. A. (1957). On a small collection of freeliving marine nematodes from Greenland and some other Arctic regions, with reviews and analyses of the compositions of all hitherto known Arctic nematode faunas. *Meddelelser om Grenland, 159*(3).
12. Allgén, C. A. (1959). Freeliving marine nematodes. *Freeliving marine nematodes.*, 1-293.
13. Angas, G. F. (1878). *Descriptions of ten Species of Marine Shells from the Province of South Australia.* Paper presented at the Proceedings of the Zoological Society of London.
14. Audouin, J. V. (1832). *Classification des Annélides, et description de celles qui habitent les côtes de la France.* Paper presented at the Annales des sciences naturelles.
15. Augener, H. (1922). Litorale Polychaeten von Juan Fernandez. *The Natural History of Juan Fernandez and Easter Island, 3*(2), 161-218.
16. Baba, K. (1976). The genus *Cerberilla* of Japan (Nudibranchia: Eolidoidea: aEolidiidae), with the description of a new species.
17. Bailey-Brock, J., & Gerstle, L. (2023). *WormLab Photograph*. Retrieved from https://www2.hawaii.edu/~wormlab/Research.html
18. Bailey-Brock, J., & Hartman, O. (1987). Class Polychaeta. *Reef and Shore Fauna of Hawaii’.(Eds DM Devaney and LG Eldredge.) pp*, 216-220.
19. Bailey-Brock, J. H. (1991). Tubeworms (Serpulidae, Polychaeta) collected from sewage outfalls, coral reefs and deep waters off the Hawaiian Islands, including a new Hydroides species. *Bulletin of Marine Science, 48*(2), 198-207.
20. Bailey-Brock, J. H., Dreyer, J., & Brock, R. E. (2003). Three new species of Saccocirrus (Polychaeta: Saccocirridae) from Hawai'i. *Pacific Science, 57*(4), 463-478.
21. Bailey-Brock, J. H., Jouin-Toulmond, C., & Brock, R. E. (2010). Protodrilidae (Annelida: Polychaeta) from the Hawaiian Islands and Comparison with Specimens from French Polynesia1. *Pacific Science, 64*(3), 463-472.
22. Baker, F., Hanna, G. D., & Strong, A. (1928). *Some Pyramidellidae from the Gulf of California*: The Academy.
23. Balk, M., & McClain, C. R. (2022). Predator-prey body size relationships in sharks. *American Naturalist, in review*.
24. Barnard, K. H. (1963). *Deep sea Mollusca from west of Cape Point, South Africa*: South African Museum.
25. Bartsch, I. (1911). The Recent and fossil mollusks of the genus *Alabina* from the west coast of America. *39*, 409-418.
26. Bartsch, P. (1911). *Recent and Fossil Mollusks of the Genus Alvania from the West Coast of America*: Smithsonian Institution Press.
27. Bartsch, P. (1912). Additions to the west American pyramidellid mollusk fauna, with descriptions of new species. *Proceedings of the United States National Museum*.
28. Bartsch, P. (1915). Report on the Turton collection of South African marine mollusks, with additional notes on other South African shells contained in the United States National Museum. *Bulletin of the United States National Museum*(91), i-305. doi:10.5479/si.03629236.91.i
29. Bartsch, P. (1917). Descriptions of new west American marine mollusks and notes on previously described forms. *Proceedings of the United States National Museum*.
30. Bartsch, P. (1920). The Caecidae and other marine mollusks from the northwest coast of America. *Journal of the Washington Academy of Sciences, 10*(20), 565-572.
31. Bartsch, P. (1922). *A monograph of the American shipworms*: US Government Printing Office.
32. Bartsch, P. (1923). Additions to our knowledge of shipworms. *Proceedings of the Biological Society of Washington*.
33. Bartsch, P. (1926). Additional new mollusks from Santa Elena Bay, Ecuador. *Proceedings of the United States National Museum, 69*(2646), 1-20. doi:10.5479/si.00963801.69-2646.1
34. Bartsch, P. (1927). New west American marine mollusks. *Proceedings of the United States National Museum, 70*(2660), 1-36. doi:10.5479/si.00963801.70-2660.1
35. Bartsch, P. (1928). New marine mollusks from Ecuador. *Journal of the Washington Academy of Sciences, 18*(3), 66-75.
36. Bebbington, A. (1974). Aplysiid species from East Africa with notes on the Indian ocean Aplysiomorpha (Gastropoda: opisthobranchia). *Zoological Journal of the Linnean Society, 54*(1), 63-99.
37. Beechey, D. (2023). The Seashells of New South Wales. Retrieved from https://seashellsofnsw.org.au/
38. Bergh, L. S. R. (1878). *Neue Chromodoriden*: T. Fischer.
39. Bergh, R. (1905). *Die Opisthobranchiata der Siboga-Expedition* (Vol. 50): Buchhandlung und druckerei vormals EJ Brill.
40. Bidgrain, P. (2020). South-west Indian Ocean Seaslug site
41. . Retrieved from http://seaslugs.free.fr/
42. Blake, J. A. (1979). Four new species of *Carazziella* (Polychaeta: Spionidae) from North and South America, with a redescription of two previously described forms.
43. Bouchet, P. (1975). Opisthobranches de profondeur de l’océan Atlantique: I–Cephalaspidea. *Cahiers de biologie marine, 16*(3), 317-365.
44. Bouchet, P. (1977). Opisthobranches de profondeur de l'océan atlantique: II. Notaspidea et Nudibranchiata. *Journal of Molluscan studies, 43*(1), 28-66.
45. Bouchet, P. (1991). New records and new species of *Abyssochrysos* (Mollusca, Caenogastropoda). *Journal of Natural History, 25*(2), 305-313. doi:10.1080/00222939100770221
46. Bouchet, P., & Kilburn, R. N. (1991). A new genus of *Ancillinae* (Mollusca, Gastropoda, Olividae) from New Caledonia, with the description of two new species. *Bulletin du Muséum National d'Histoire Naturelle, Section A (4), 12*, 531-539.
47. Boughet, P., & Warén, A. (1979). The abyssal molluscan fauna of the Norwegian Sea and its relation to other faunas. *Sarsia, 64*(3), 211-243.
48. Brazier, J. (1877). *Shells collected during the Chevert Expedition.* Paper presented at the Proc. Linn. Soc. NSW.
49. Brazier, J. (1894). *List of Mollusca found at Green Point, Watson’s Bay, Sydney, by Arnold U. Henn. With a few remarks on some of the most interesting species and descriptions of the new species by John Brazier.* Paper presented at the Proceedings of the Linnean Society of New South Wales, series.
50. Breslau, E., Valdés, Á., & Chichvarkhin, A. (2016). A new cryptic species of *Melanochlamys* (Gastropoda: Heterobranchia: Cephalaspidea) from the Northwestern Pacific. *American Malacological Bulletin, 34*(2), 103-108.
51. Brown, T. (1827). Illustrations of the conchology of Great Britain and Ireland.
52. Burn, R. (1969). Descriptions of Australian Eolidacea (Mollusca: Opisthobranchia) 4. The genera Pleurolidia, Fiona, Learchis and Cerberilla from Lord Howe Island. *Journal of the Malacological Society of Australia, 1*(10), 21-34.
53. Burn, R. (2015). *Nudibranchs and related molluscs*: Museum Victoria.
54. Caballer, M., Ortea, J., & Redfern, C. (2014). On the Genus *Rissoella* Gray, 1847 (Gastropoda: Heterobranchia: Rissoellidae) in the Bahamas. *American Malacological Bulletin, 32*(1), 104-121. doi:10.4003/006.032.0109
55. Carmona, L., Bhave, V., Salunkhe, R., Pola, M., Gosliner, T. M., & Cervera, J. L. (2014). Systematics of Anteaeolidiella. *Zoological Journal of the Linnean Society, 171*(1), 108-132. doi:10.1111/zoj.12129
56. Carmona, L., Lei, B. R., Pola, M., Gosliner, T. M., Valdés, Á., & Cervera, J. L. (2014). The ‘Spurilla Neapolitana’ Species Complex. *Zoological Journal of the Linnean Society, 170*(1), 132-154. doi:10.1111/zoj.12098
57. Carmona, L., Pola, M., Gosliner, T. M., & Cervera, J. L. (2014a). The Atlantic-Mediterranean genus *Berghia* Trinchese, 1877 (Nudibranchia: Aeolidiidae): taxonomic review and phylogenetic analysis. *Journal of Molluscan studies, 80*(5), 482-498. doi:10.1093/mollus/eyu031
58. Carmona, L., Pola, M., Gosliner, T. M., & Cervera, J. L. (2014b). The end of a long controversy: systematics of the genus *Limenandra* (Mollusca: Nudibranchia: Aeolidiidae). *Helgoland Marine Research, 68*(1), 37-48. doi:10.1007/s10152-013-0367-y
59. Carmona, L., Pola, M., Gosliner, T. M., & Cervera, J. L. (2017). Integrative taxonomy and biogeography of the genus *Bulbaeolidia* (Nudibranchia: Aeolidida). *Journal of Molluscan studies, 83*(4), 440-450.
60. Carpenter, P. P. (1866). XXIX.—On the Pleistocene fossils collected by Col. E. Jewett at Sta. Barbara (California); with descriptions of new species. *Annals and Magazine of Natural History, 17*(100), 274-278.
61. Cavallari, D. C., Almeida, S. M., & Simone, L. R. L. (2020). Cerithiidae, Litiopidae, Modulidae and Planaxidae (Gastropoda, Cerithioidea) collected by the Marion Dufresne MD55 expedition in southeastern Brazil. *Papéis Avulsos de Zoologia, 60*.
62. Cavallari, D. C., Salvador, R. B., Dornellas, A. P., & Simone, L. R. (2019). Calliostomatidae, Colloniidae, Margaritidae, and Solariellidae (Gastropoda: Trochoidea) collected by the Marion Dufresne (MD55) expedition in southeastern Brazil, with description of a new species of *Calliostoma*. *Zootaxa, 4609*(3), 401-428.
63. Cavallari, D. C., Salvador, R. B., & Simone, L. R. (2014). Taxonomical study on the Architectonicidae collected by the Marion Dufresne (MD55) expedition to SE Brazil. *Spixiana, 37*(1), 35-43.
64. Cheeseman, T. (1881). *On some new species of nudibranchiate Mollusca.* Paper presented at the Transactions and Proceedings of the New Zealand Institute.
65. Chichvarkhin, A. (2016). Shallow water sea slugs (Gastropoda: Heterobranchia) from the northwestern coast of the Sea of Japan, north of Peter the Great Bay, Russia. *PeerJ, 4*, e2774.
66. Clapp, W. (1924). *Three new species of Teredo* (Vol. 25).
67. Clapp, W. F. (1923). *A new species of Teredo from Florida*.
68. Clarke Jr, A. H., & Menzies, R. J. (1959). Neopilina (Vema) ewingi, a second living species of the Paleozoic class Monoplacophora. *Science, 129*(3355), 1026-1027.
69. Clench, W., & Turner, R. (1946). The genus *Bankia* in the western Atlantic. *Johnsonia, 2*(19), 1-28.
70. Clench, W. J. (Ed.) (1946-1953). *Johnsonia: Monographs of the Marine Mollusks of the Western Atlantic* (Vol. II (19-32)). Cambridge, Massachusetts: Botantical Museum of Harvard University.
71. Clench, W. J. (Ed.) (1960-1970). *Johnsonia: Monographs of the Marine Mollusks of the Western Atlantic* (Vol. IV (40-48)). Cambridge, Massachusetts: Botantical Museum of Harvard University.
72. Connolly, M. W. K. (1939). *A monographic survey of South African non-marine Mollusca*: Trustees of the South African Museum.
73. Cooke, S., Hanson, D., Hirano, Y., Ornelas‐Gatdula, E., Gosliner, T. M., Chernyshev, A. V., & Valdés, Á. (2014). Cryptic diversity of Melanochlamys sea slugs (Gastropoda, Aglajidae) in the North Pacific. *Zoologica Scripta, 43*(4), 351-369. doi:10.1111/zsc.12063
74. Corgan, J. X. (1972). Pacific species of *Nesiodostomia* Pilsbry, 1918, and Puposyrnola Cossmann, 1921. *Veliger, 14*(4), 355-360.
75. Crane, J. (1975). *Fiddler Crabs of the World: Ocypodidae: Genus Uca* (Vol. 1276): Princeton University Press.
76. Cunha, C. M., & Simone, L. R. L. (2018). A new species of the genus *Rapturella* (Gastropoda: Acteonidae) from southeast Brazil. *Zootaxa, 4521*(1), 125-128. doi:10.11646/zootaxa.4521.1.7 PMID - 30486165
77. Dall, W. H. (1908). *The mollusca and the brachiopoda*: Harvard College, Museum of Comparative Zoology.
78. Dall, W. H. (1908). Reports on the dredging operations off the west coast of Central America to the Galapagos, to the west coast of Mexico, and in the Gulf of California, in charge of Alexander Agassiz, carried on by the US Fish Commission steamer "Albatross", during 1891, Lieut. Commander ZL Tanner, USN, commanding. XXXVII. Reports on the scientific results on the expedition to the eastern tropical Pacific in charge of Alexander Agassiz, by the US Fish Commission steamer" Albatross", from October, 1904, to March, 1905, Lieut. Commander LM Garrett, USN, commanding. XIV. The Mollusca and the Brachiopoda. *Bull. Mus. Comp. Zool., 43*(6), 205-487, 219pls.
79. Dall, W. H. (1913). New species of the genus *Mohnia* from the North Pacific. *Proceedings of the Academy of natural sciences of Philadelphia*, 501-504.
80. Dall, W. H. (1919). Descriptions of new species of Mollusca from the North Pacific Ocean in the collection of the United States National Museum. *Proceedings of the United States National Museum*.
81. Dall, W. H., & Bartsch, P. (1904). Synopsis of the genera, subgenera, and sections of the family Pyramidellidae.
82. Dall, W. H., & Bartsch, P. (1906). Notes on Japanese, Indopacific, and American Pyramidellidae. *Proceedings of the United States National Museum*.
83. Dall, W. H., & Bartsch, P. (1909). *A monograph of west American pyramidellid mollusks*: US Government Printing Office.
84. Dautzenberg, P., I, A., Fischer, H., & de Boury, E. (1896). *Campagnes scientifiques de SA le Prince Albert Ier de Monaco: Dragages effectués par l'Hirondelle et par la Princesse-Alice, 1888-1895*.
85. Day, J. H. (1961). The polychaet fauna of South Africa. Part 6. Sedentary species dredged off Cape coasts with a few new records from the shore. *Zoological Journal of the Linnean Society, 44*(299), 463-560.
86. Day, J. H. (1967). A monograph on the Polychaeta of southern Africa. Part 2. Sedentaria. *British Museum (Natural History) Publications, 656*, 459-878.
87. de La Saussaye, S. P. (1869). *Catalogue des mollusques testacés des mers d'Europe*: F. Savy.
88. Deel, I. (2000). Caecidae (Mollusca: Gastropoda) from Mauritania. *INTERNATIONAL MAGAZINE ON SEA AND SHELLS, 47*(3), 81.
89. Dell, R. (1956). Some new off-shore Mollusca from New Zealand. *Records of the Dominion Museum, 3*(1), 27-59.
90. Di Geronimo, I., Privitera, S., & Valdovinos, C. (1995). Fartulum magellanicum (Prosobranchia, Caecidae): a new species from the Magellanic Province. *Bol. Soc. Biol. Concepción, Chile, 66*, 115-120.
91. Dornellas, A. P. S. (2012). Description of a new species of Calliostoma (Gastropoda, Calliostomatidae) from Southeastern Brazil. *ZooKeys*(224), 89.
92. Edmunds, M. (2015). Opisthobranchiate Mollusca from Ghana: Aeolidiidae, with consideration of several Caribbean species. *Journal of Conchology, 42*(1), 1.
93. Eliot, C. (1903). Notes on some new or little-known members of the family Doridiidae. *Journal of Molluscan studies, 5*(5), 331-337.
94. Ellis, B. F., & Messina, A. R. (1940). Catalogue of foraminifera. *(No Title)*.
95. Ellis, B. F., & Messina, A. R. (1952). *Catalogue of Ostracoda*: American Museum of Natural History.
96. *Encylopedia of Life*. (2022). Retrieved from: http://eol.org
97. Espinosa, J., & Ortea, J. (2011). Nuevas especies de moluscos gasterópodos (Mollusca: Gastropoda), con caracteres singulares, recolectadas en las cuevas submarinas de Cuba. *22*(4), 150-160.
98. Fahey, S. J., & Gosliner, T. M. (2004). A phylogenetic analysis of the Aegiridae Fischer, 1883 (Mollusca, Nudibranchia, Phanerobranchia) with descriptions of eight new species and a reassessment of phanerobranch relationships. *Proceedings-California Academy Of Sciences, 55*(26/35), 613.
99. Fauchald, K., Granados-Barba, A., & Solís-Weiss, V. (2009). Polychaeta (Annelida) of the Gulf of Mexico. *Gulf of Mexico Origin, Waters, and Biota, 1*, 751-788.
100. Filip, E. (1969). Free-living marine nematodes of the Sevastopol area. *Free-living marine nematodes of the Sevastopol area.*
101. Filipʹev, I. N. (1973). Free living nematodes in the collection of the Zoological Museum of the Imperial Academy of Sciences in Petrograd= Svobodnozhivushchie nematody kolektsii zoologicheskogo muzeya Imperatorskoi Akademii Nauk v Petrograd. *(No Title)*.
102. Fisher, W. K. (1952). The Sipunculid Worms of California and Baja California. *Proceedings of the United States National Museum, 102*(3306), 371-450, plates 318-339. doi:10.5479/si.00963801.102-3306.371
103. Froese, R., & Pauly, D. (2022). Fishbase: a global information system on fishes.
104. García-Gómez, J., Cimino, G., & Medina, A. (1990). Studies on the defensive behaviour of Hypselodoris species (Gastropoda: Nudibranchia): ultrastructure and chemical analysis of mantle dermal formations (MDFs). *Marine Biology, 106*, 245-250.
105. Garrett, A. (1873). Descriptions of new species of marine shells inhabiting the South Sea Islands. *Proceedings of the Academy of natural sciences of Philadelphia*, 209-231.
106. Gerlach, S. A. (1953). *Freilebende marine Nematoden aus dem Küstengrundwasser und aus dem Brackwasser der chilenischen Küste*: Gleerup.
107. Giere, O., Ebbe, B., & Erséus, C. (2008). Questa (Annelida, Polychaeta, Orbiniidae) from Pacific regions—new species and reassessment of the genus Periquesta. *Organisms Diversity & Evolution, 7*(4), 304-319.
108. Gofas, S., & Hoffman, L. (2020). Deep-water Calliostomatidae (Vetigastropoda, Gastropoda) from the South Azorean Seamount Chain. *Iberus, 38*(2), 195-211.
109. Gofas, S., Luque, Á. A., Oliver, J. D., Templado, J., & Serrano, A. (2021). The Mollusca of Galicia Bank (NE Atlantic Ocean). *European Journal of Taxonomy, 785*, 1–114-111–114. doi:10.5852/ejt.2021.785.1605
110. Goodey, T. (1963). Soil and freshwater nematodes. A monograph. *Soil and freshwater nematodes. A monograph.*(Edn 2).
111. Gosliner, T. (2011). Six new species of aglajid opisthobranch mollusks from the tropical Indo-Pacific. *Zootaxa, 2751*(1), 1-24. doi:10.11646/zootaxa.2751.1.1
112. Gosliner, T. (2015). Three new species of aglajid Cephalaspidean mollusks from the tropical Indo-pacific of the Verde Island passage. *Proceedings of the California Academy of Sciences, 6*, 191-205.
113. Gosliner, T. M. (1985). The aeolid nudibranch family Aeolidiidae (Gastropoda: Opisthobranchia) from tropical southern Africa. *Annals of the South African Museum, 95*, 233-267.
114. Gosliner, T. M. (1990). Sleuthing cryptic Chromodorids (Mollusca, Nudibranchia): adding to Philippine marine biodiversity.
115. Gosliner, T. M. (2010). Two new species of nudibranch mollusks from the coast of California. *Proceedings of the California Academy of Sciences, 61*(7), 623.
116. Gosliner, T. M., & Johnson, S. (1994). Review of the genus *Hallaxa* (Nudibranchia: Actinocyclidae) with descriptions of nine new species. *37*(2), 155-191.
117. Gould, A. A. (1849). *Descriptions of new species of shells, brought home by the US Exploring Expedition.* Paper presented at the Proceedings of the Boston Society of Natural History.
118. Gould, A. A. (1852). United States Exploring Expedition during the Years 1838–1842 under the Command of Charles Wilkes, USN Volume 12. Mollusca and Shells. In: Gould & Lincoln, Boston. Available from http://biodiversitylibrary. org/page ….
119. Gould, A. A. (1861). Description of new shells collected by the United States North Pacific Exploring Expedition. *Proceedings of the Boston Society of Natural History, 7*, 385-389.
120. Gracia, A., Adrila, N. E., & Diaz, J. M. (2004). Gastropods collected along the continental slope of the Colombian Caribbean during the INVEMAR-Macrofauna campaigns (1998-2001). *Iberus, 22*, 43-75.
121. Güller, M., & Zelaya, D. G. (2019). Revision of Pyramidellidae (Gastropoda: Heterobranchia) from Argentina triples their diversity in northern Patagonia. *Journal of Molluscan studies, 85*(1), 103-125.
122. Habe, T. (1952). Parasitic gastropods found in echinoderms from Japan. *Publications of the Seto Marine Biological Laboratory, 2*(2), 73-85. doi:10.5134/174685
123. Habe, T. (1958a). The Fauna of Akkeshi Bay: XXV. Gastropoda (With Plates IV). *8*, 2-39.
124. Habe, T. (1958b). On the shell-bearing opisthobranchiate molluscan fauna from off Choshi, Chiba Pref., Japan. *日本動物学彙報, 31*(2), 117-120.
125. Habe, T. (1976). Eight new and little known cephalaspid Opisthobranchia from Japan. *Venus (Japanese Journal of Malacology), 35*(4), 151-157.
126. Hartman, O. (1960). Systematic account of some marine invertebrate animals from the deep basins off southern California. *Allan Hancock Pacific Expeditions, 22*(2), 69-176.
127. Hartmann-Schröder, G. (1979). Die Polychaeten der tropischen Nordwestküste Australiens (zwischen Derby im Norden und Port Hedland im Süden). *Mitteilungen aus dem Hamburgischen zoologischen Museum und Institut, 76*, 75-218.
128. Hedley, C. (1906). *The Mollusca of Mast Head Reef, Capricorn Group, Queensland.* Paper presented at the Proceedings of the Linnean Society of New South Wales.
129. Hedley, C. (1907). The results of deep-sea investigation in the Tasman Sea. II. The expedition of the ‘Woy Woy.’. *Records of the Australian Museum, 6*, 356-364.
130. Hedley, C. (1912). *Descriptions of some new or noteworthy shells in the Australian Museum*: Australian Museum.
131. Hertlein, L., & Strong, A. (1951). Eastern Pacific expeditions of the New York Zoological Society. XLIII. Mollusks from the west coast of Mexico and Central America. Part X. *New York Zoological Society, Zoologica, 36*(2), 66-120.
132. Hertz, J. (1994). Review of the type species of Lirobarleeia Ponder, 1983. *The Veliger, 37*(1), 110-116.
133. Hoffman, L., Gofas, S., & Freiwald, A. (2020). A large biodiversity of “skeneimorph”(Gastropoda: Vetigastropoda) species from the South Azorean Seamount Chain, with the description of seventeen new species. *38*(Suplemento 9), 1-82.
134. Høisæter, T. (2014). The Pyramidellidae (Gastropoda, Heterobranchia) of Norway and adjacent waters. A taxonomic review. *Fauna norvegica, 34*(0), 7-78. doi:10.5324/fn.v34i0.1672
135. Hori, S., & Fukuda, H. (1999). New Species of the Pyramidellidae (Orthogastropoda : Heterobranchia) from the Collections of the Yamaguchi Museum and the Hagi City Museum. *Venus (Japanese Journal of Malacology), 58*(4), 175-190. doi:10.18941/venusjjm.58.4_175
136. Hornung, A., & Mermod, G. (1924). Mollusques de la Mer Rouge recueillis par A. Issel faisant partie des collections du Musée Civique d’Histoire Naturelle de Gênes. Première partie, Pyramidellides. *Annali del Museo Civico di Storia Naturale “G. Doria, 51*, 283-311.
137. Houbrick, R. S. (1980). Review of the deep-sea genus *Argyropeza* (Gastropoda: Prosobranchia: Cerithiidae).
138. Houbrick, R. S. (1992). Monograph of the genus *Cerithium* Bruguière in the Indo-Pacific (Cerithiidae: Prosobranchia).
139. Hutton, F. W. (1885). Descriptions of new Tertiary shells.
140. Hylleberg, J. (2013). Classification and identificion of sipunculans from Thailand, with descruption of new species and a new subgenus. *Phuket Marine Biological Center Special Publication, 32*, 53-82.
141. Imajima, M. (1972). Review of the annelid worms of the family Nereidae of Japan, with descriptions of five new species or subspecies. *Bulletin of National Science Museum, 15*, 37-153.
142. Iredale, T. (1912). New generic names and new species of marine Mollusca. *Journal of Molluscan studies, 10*(3), 217-228.
143. Iredale, T. (1936). Australian molluscan notes. No. 2. *Records of the Australian Museum, 19*(5), 267-340. doi:10.3853/j.0067-1975.19.1936.704
144. Issel, A. (1869). *Malacologia del Mar Rosso: ricerche zoologiche e paleontologiche*: Biblioteca malacologia.
145. Jeffreys, J. G. (1860). XXII.—A synoptical list of the British species of *Teredo,* with a notice of the exotic species. *Annals and Magazine of Natural History, 6*(32), 121-127.
146. Jeffreys, J. G. (1883). XLIX.—Mediterranean Mollusca (No. 3) and other invertebrata. *Journal of Natural History, 11*(66), 393-401.
147. Kano, Y., Chikyu, E., & Warén, A. (2009). Morphological, ecological and molecular characterization of the enigmatic planispiral snail genus *Adeuomphalus* (Vetigastropoda: Seguenzioidea). *Journal of Molluscan studies, 75*(4), 397-418. doi:10.1093/mollus/eyp037
148. Kantor, Y. I., Harasewych, M. G., & Puillandre, N. (2016). A critical review of Antarctic Conoidea (Neogastropoda). *Molluscan Research, 36*(3), 153-206.
149. Kantor, Y. I., Kosyan, A., Sorokin, P., Herbert, D. G., & Fedosov, A. (2020). Review of the abysso-hadal genus *Bayerius* (Gastropoda: Neogastropoda: Buccinidae) from the north-west Pacific, with description of two new species. *Deep Sea Research Part I: Oceanographic Research Papers, 160*, 103256.
150. Kay, A. E. (1979). Hawaiian marine shells. Reef and shore fauna of Hawaii: Section 4: Mollusca. *BP Bishop Museum Special Publication, 64*(4), 1-653.
151. Keen, A. M. (1971). Sea shells of tropical West America.
152. Kienberger, K., Carmona, L., Pola, M., Padula, V., Gosliner, T. M., & Cervera, J. L. (2016). Aeolidia papillosa (Linnaeus, 1761) (Mollusca: Heterobranchia: Nudibranchia), single species or a cryptic species complex? A morphological and molecular study. *Zoological Journal of the Linnean Society, 177*(3), 481-506. doi:10.1111/zoj.12379
153. Kilburn, R. (1973). Notes on some benthic Mollusca from Natal and Mocambique, with descriptions of new species and subspecies of *Calliostoma, Solariella, Latiaxis, Babylonia, Fusinus, Baythytoma* and *Conus*. *Annals of the Natal Museum, 21*(3), 557-578.
154. Killeen, I. J., & Oliver, P. G. (2000). A new species of *Abyssochrysos* (Gastropoda: Loxonematoidea) from the Oman margin. *Journal of Molluscan studies, 66*(1), 95-98. doi:10.1093/mollus/66.1.95
155. Kirkegaard, J. B. (1959). *The Polychaeta of West Africa*.
156. Knudsen, J. (1964). Scaphopoda and Gastropoda from depths exceeding 6000 meters. *Galathea Report, 7*, 125-136.
157. Korshunova, T., Martynov, A., Bakken, T., Evertsen, J., Fletcher, K., Mudianta, I. W., . . . Picton, B. (2017). Polyphyly of the traditional family Flabellinidae affects a major group of Nudibranchia: aeolidacean taxonomic reassessment with descriptions of several new families, genera, and species (Mollusca, Gastropoda). *ZooKeys, 717*(717), 1-139. doi:10.3897/zookeys.717.21885 PMID - 29391848
158. Korshunova, T., Zimina, O., & Martynov, A. (2017). Unique pleuroproctic taxa of the nudibranch family Aeolidiidae from the Atlantic and Pacific Oceans, with description of a new genus and species. *Journal of Molluscan studies, 83*(4), 409-421. doi:10.1093/mollus/eyx036
159. Kramp, P. L. (1961). Synopsis of the Medusae of the World. *Journal of the marine biological Association of the United Kingdom, 40*, 7-382. doi:10.1017/s0025315400007347
160. Krug, P. J., Morley, M. S., Asif, J., Hellyar, L. L., & Blom, W. M. (2008). Molecular confirmation of species status for the rare cephalaspidean *Melanochlamys lorrainae* (Rudman, 1968), and comparison with its sister species *M. cylindrica* Cheeseman, 1881. *Journal of Molluscan studies, 74*(3), 267-276. doi:10.1093/mollus/eyn018
161. Ladd, H. S. (1966). *Chitons and gastropods (Haliotidae through Adeorbidae) from the western Pacific islands* (Vol. 531): US Government Printing Office.
162. Langerhans, P. (1881). *Ueber einige canarische Anneliden* (Vol. 42): Blochmann.
163. Laseron, C. F. (1951). The New South Wales Pyramidellidae and the genus *Mathilda*. *Records of the Australian Museum, 22*(4), 298-334. doi:10.3853/j.0067-1975.22.1951.610
164. Laseron, C. F. (1954). Revision of the Liotiidae of New South Wales. *Australian Zoologist, 12*(1), 1-25.
165. Laseron, C. F. (1958). *Liotiidae and allied molluscs from the Dampierian Zoogeographical Province*: Records of Australian Museum.
166. Laseron, C. F. (1959). The Family Pyramidellidae (Mollesca) from Northern Australia. *Marine and Freshwater Research, 10*(2), 177-268. doi:10.1071/mf9590177
167. Laws, C. R. (1937, 1937). *Review of the Tertiary and Recent Neozelanic pyramidellid molluscs. No. 2. The genus Chemnitzia*.
168. Laws, C. R. (1941, 1941). *Review of the Tertiary and Recent Neozelanic Pyramidellid molluscs. No. 8—The pyrgulinid genera and the genus Evalea*.
169. Liggia, B. (2013). *Worldwide Mollusc Speces Data Base*. Retrieved from: https://www.bagniliggia.it/WMSD/WMSDhome.htm
170. Lima, S. F. B., Simone, L. R. L., & Guimarães, C. R. P. (2016). Addisonia enodis (Vetigastropoda: Lepetelloidea) associated with an elasmobranch egg capsule from the South Atlantic Ocean and the discovery of the species from deep waters off northeastern Brazil. *Biota Neotropica, 16*(3), e20160202. doi:10.1590/1676-0611-bn-2016-0202
171. Linden, J. V. D., & Eikenboom, J. C. A. (1992). On the taxonomy of the Recent species of the genus *Chrysallida* Carpenter from Europe, the Canary Islands and the Azores (Gastropoda, Pyramidellidae). *56*(1/3), 3-63.
172. Liow, L. H., & Taylor, P. D. (2019). Cope's Rule in a modular organism: Directional evolution without an overarching macroevolutionary trend. *Evolution, 73*(9), 1863-1872.
173. Locard, A. (1897). Expéditions scientifiques du Travailleur et du Talisman pendant les années 1880, 1881, 1882, 1883. Mollusques testaces. *(No Title)*.
174. MagalhÃes, W. F., Bailey-Brock, J., & Watling, L. (2018). Four new species of *Magelona* (Annelida: Magelonidae) from Easter Island, Guam and Hawaii. *Zootaxa, 4457*(3), 379-396.
175. Magalhaes, W. F., Bailey-Brock, J. H., & Rizzo, A. E. (2012). Lacydonia quadrioculata, a new lacydoniid (Polychaeta: Phyllodocida) from Oahu, Hawaii. *Zootaxa, 3589*(1), 65–76-65–76.
176. Magalhães, W. F., Bailey-Brock, J. H., & Santos, C. S. (2015). A new species and two new records of *Poecilochaetus* (Polychaeta: Poecilochaetidae) from Hawaii. *Journal of the marine biological Association of the United Kingdom, 95*(1), 91-100.
177. Magalhaes, W. F., Bailey–Brock, J. H., & Davenport, J. S. (2011). On the genus Raphidrilus Monticelli, 1910 (Polychaeta: Ctenodrilidae) with description of two new species. *Zootaxa, 2804*(1), 1–14-11–14.
178. Marincovich, L. (1973). Intertidal mollusks of Iquique, Chile.
179. Marshall, B. (1979). The Trochidae and Turbinidae of the Kermadec Ridge (Mollusca: Gastropoda). *New Zealand Journal of Zoology, 6*(4), 521-552.
180. Marshall, B. (1988). Thysanodontinae: a new subfamily of the Trochidae (Gastropoda). *Journal of Molluscan studies, 54*(2), 215-229.
181. Marshall, B. A. (1988). Skeneidae, Vitrinellidae and Orbitestellidae (Mollusca: Gastropoda) associated with biogenic substrata from bathyal depths off New Zealand and New South Wales. *Journal of Natural History, 22*(4), 949-1004. doi:10.1080/00222938800770631
182. Marshall, B. A. (1996). A new subfamily of the Addisoniidae associated with cephalopod beaks from the tropical southwest Pacific, and a new pseudococculinid associated with chondrichthyan egg cases from New Zealand (Mollusca: Lepetelloidea). *Veliger, 39*(3), 250-259.
183. Marshall, B. A., & Walton, K. (2019). A review of Buccipagoda Ponder, 2010 and descriptions of new species and a new genus (Gastropoda: Neogastropoda: Buccinoidea: Buccinidae). *Molluscan Research, 39*(1), 70-81.
184. McClain, C. R., Gullett, T., Jackson‐Ricketts, J., & Unmack, P. J. (2012). Increased energy promotes size‐based niche availability in marine mollusks. *Evolution: International Journal of Organic Evolution, 66*(7), 2204-2215.
185. McLean, J. H., & Andrade, H. (1982). *Large archibenthal gastropods of central Chile: collections from an expedition of the R/V Anton Bruun and the Chilean shrimp fishery*: Natural History Museum of Los Angeles County.
186. Mehrotra, R., Gutiérrez, M. A. C., Scott, C. M., Arnold, S., Monchanin, C., Viyakarn, V., & Chavanich, S. (2021). An updated inventory of sea slugs from Koh Tao, Thailand, with notes on their ecology and a dramatic biodiversity increase for Thai waters. *ZooKeys, 1042*, 73.
187. Mello, R. d. L. S., & Maestrati, P. (1986). A família Caecidae Gray, 1850 no nordeste do Brasil.
188. Melvill, J. C. (1896). Descriptions of new species of minute marine shells from Bombay. *Journal of Molluscan studies, 2*(3), 108-116.
189. Melvill, J. C. (1906). Descriptions of thirty-one Gastropoda and one scaphopod from the Persian Gulf and Gulf of Oman, dredged by Mr. FW Townsend, 1902–1904. *Journal of Molluscan studies, 7*(2), 69-80.
190. Melvill, J. C. (1910). A revision of the species of the family Pyramidellidæ occurring in the Persian Gulf,
191. Gulf of Oman, and North Arabian Sea, as exemplified mostly in the collections made by Mr. FW townsend (1893–1900), with descriptions of new species. *Journal of Molluscan studies, 9*(3), 171-207. doi:10.1093/oxfordjournals.mollus.a066331
192. Merle, D., Garrigues, B., & Pointier, J.-P. (2011). *Fossil and recent Muricidae of the world: part Muricinae*: ConchBooks.
193. Miller, M. C. (1987). Hallaxa gilva, a new dorid nudibranch (Gastropoda: Opisthobranchia) from New Zealand. *New Zealand Journal of Zoology, 14*(1), 123-129. doi:10.1080/03014223.1987.10422689
194. Miller, M. C. (2001). Aeolid nudibranchs (Gastropoda: Opisthobranchia) of the family Aeolidiidae from New Zealand waters. *Journal of Natural History, 35*(5), 629-662. doi:10.1080/00222930152023081
195. Mimoto, K., & Nakao, K. i. (2009). Newly found molluscan species from the Ananai Formation of the Plio-Pleistocene Tonohama Group in Kochi Prefecture, Japan: Part 9. *徳島県立博物館研究報告*(30), 15-25.
196. Moore, D. R. (1972). Ecological and systematic notes on Caecidae from St. Croix, US Virgin Islands. *Bulletin of Marine Science, 22*(4), 881-899.
197. Moore, R. C., & Pitrat, C. W. (1961). *Treatise on Invertebrate Paleontology. Part Q, Arthropoda 3: Crustacea, Ostracoda.* . Boulder, Colorado: Geological Society of America.
198. Mörch, O. A. L. (1863). *Contributions à la faune malacologique des Antilles Danoises*: Imprimerie de Madame Veuve Bouchard-Huzard, rue de l'Eperon, 5.
199. Moro, L., & Ortea, J. (2015). Nuevos taxones de babosas marinas de las islas Canarias y de Cabo Verde (Mollusca: Heterobranchia). *Vieraea, 43*, 21-86.
200. Murina, V. V. (1968). New species of Sipuncula from the Red Sea. *Zoologicheskii Zhurnal, 47*, 1722-1725.
201. Nomura, S. (1936). Pyramidellidae from Siogama Bay, northeast Honsyu, Japan. *Saito Ho-on Kai Mus. Res. Bull., 10*, pls-1.
202. O’Donoghue, C. (1929). Opisthobranchiate Mollusca collected by the South African marine biological survey. *Reports of Fisheries and Marine Biological Survey, Union of South Africa, 7*, 1-84.
203. Okutani, T., & Fujikura, K. (2002). Abyssal gastropods and bivalves collected by Shinkai 6500 on slope of the Japan Trench. *Venus (Journal of the Malacological Society of Japan), 60*(4), 211-224.
204. Ortea, J., Luque, A. A., & Templado, J. (1990). Contributions to the knowledge of the Genus *Aegires* Lovén, 1844 (Opisthobranchia: Doridoidea: Aegiretidae) in the North Atlantic, with descriptions of two new species. *Journal of Molluscan Studies, 56*(3), 333-337. doi:10.1093/mollus/56.3.333
205. Ortea, J., & Moro, L. (2009). Descripción de una nueva especie del género Elysia Risso, 1818 (Mollusca: Sacoglossa) recolectada en las islas Canarias, nombrada en honor de César Manrique. *Vieraea, 37*, 91-98.
206. Ortea, J., & Moro, L. (2018). Descripción de una nueva especie de Aglajidae Pilsbry, 1895 (Mollusca: Cephalaspidea) de las islas de Cabo Verde, con algunas consideraciones sobre la ordenación taxonómica de la familia. In: Avicennia.
207. Ortea, J., Moro, L., & Bacallado, J. (2006). Ubicación de Baptodoris perezi Llera & Ortea, 1982 en el género Gargamella Bergh, 1894 (Mollusca: Nudibranchia). *Vieraea, 34*, 55-58.
208. Ortea, J., Moro, L., Bacallado, J. J., & Caballer, M. (2014). Nuevas especies y primeras citas de babosas marinas (Mollusca: Opisthobranchia) en las islas Canarias y en otros archipiélagos de la Macaronesia. *Vieraea Folia scientiarum biologicarum canariensium, 42*(Vieraea 42), 47-77. doi:10.31939/vieraea.2014.42.04
209. Ortea, J., Moro, L., Caballer, M., & Hernández, F. (2003). Nota sobre la propuesta de sinonimia de *Tyrinna burnayi* (Ortea, 1988) de las islas de Cabo Verde con Tyrinna evelinae (Marcus, 1958) del Brasil (Mollusca: Opisthobranchia: Chromodorididae). *Revista de la Academia Canaria de Ciencias, 15*, 191-196.
210. Ortea, J., Moro, L., & Espinosa, J. (1996). Descripción de dos nuevas especies del género *Chelidonura* A. Adams, 1850 (Opisthobranchia: Cephalaspidea: Aglajidae) colectadas en la Isla de El Hierro. Estudio comparado con C. africana Pruvot-Fol 1953. *Revista de la Academia Canaria de Ciencias, 7*(2), 3.
211. Ortea, J., Moro, L., & Espinosa, J. (2015). Estudio de un grupo de especies caribeñas enmascaradas en el nombre *Aegires sublaevis* Odhner, 1932 (Mollusca: Nudibranchia) utilizando técnicas tradicionales. *Revista Academia Canaria de Ciencias*, 243-258.
212. Ortea, J., Valdés, Á., & García-Gómez, J. C. (1996). *Revisión de las especies atlánticas de la familia Chromodorididae (Mollusca: Nudibranchia) del grupo cromático azul: Review of the atlantic species of the family Chromodorididae (Mollusca: Nudibranchia) of the blue chromatic group*: Universidad de Oviedo.
213. Oug, E. (1978). New and lesser known Dorvilleidae (Annelida, Polychaeta) from Scandinavian and northeast American waters. *Sarsia, 63*(4), 285-303.
214. Padula, V., & Delgado, M. (2010). A new species of *Cerberilla* (Gastropoda: Nudibranchia: Aeolidiidae) from northeastern Brazil. *Nautilus, 124*(4), 175.
215. Palomares, M. L. D., & Paulay, G. (2022). *SeaLifeBase*. Retrieved from: www.sealifebase.org
216. Paramonov, A. A. (1976). *Plant parasite nematodes* (Vol. 2): Indian National Scientific Documentation Centre,[available from the US ….
217. Pastorino, R. S. G., & Chiesa, I. L. (2014). The family Caecidae (Gastropoda: caenogastropoda) in Argentine waters.
218. Pease, W. (1871). Descriptions of nudibranchiate Mollusca inhabiting Polynesia. *American Journal of Conchology, 6*(4), 299-305.
219. Pelorce, J., Horst, D., & Hoarau, A. (2013). Une nouvelle espèce de la famille Aglajidae (Gastropoda: Opisthobranchia) des côtes de Méditerranée française. *Iberus, 31*(2), 165-170.
220. Peñas, A., & Rolán, E. (1997). *La familia Pyramidellidae Gray, 1840 (Mollusca, Gastropoda, Heterostropha) en África Occidental. 2. Los géneros Turbonilla y Eulimella*: Sociedad Española de Malacología.
221. Peñas, A., & Rolán, E. (1998). La familia Pyramidellidae Gray, 1840 (Mollusca, Gastropoda, Heterostropha) en África occidental. 3. *El género Chrysallida sl Iberus, Supplement, 4*, 1-73.
222. Peñas, A., & Rolán, E. (1999). La familia Pyramidellidae Gray, 1840 (Mollusca, Gastropoda, Heterostropha) en África Occidental. 4. Los géneros Megastomia, Odostomia, Ondina, Noemiamea y Syrnola. *Iberus, suplemento, 5*(1), 150.
223. Peñas, A., & Rolán, E. (2002). La superfamilia Pyramidelloidea Gray, 1840 (Mollusca, Gastropoda, Heterostropha) en África Occidental. 10. Addenda 2. *Iberus, 20*(1), 1-54.
224. Peñas, A., & Rolán, E. (2017). *Deep water Pyramidelloidea from the Central and South Pacific: the tribe Chrysallidini*.
225. Peñas, A., Rolán, E., & Swinnen, F. (2014). The superfamily Pyramidelloidea Gray, 1840 (Mollusca, Gastropoda, Heterobranchia) in West Africa, 11. Addenda 3. In: Iberus.
226. Perkins, T. H. (1981). Syllidae (Polychaeta), principally from Florida, with descriptions of a new genus and twenty-one new species. *Proc. Biol. Soc. Wash, 93*(4), 1080-1172.
227. Perrone, A. S. (1990). Una nuova specie di Aglajidae dal fondo batiale del Golfo di Taranto: Chelidonura orchidaea nov. sp.(Opisthobranchia: Philinoidea). *Bollettino Malacologico, 26*, 105-112.
228. Pettibone, M. H. (1963). Marine polychaete worms of the New England region. I. Aphroditidae through Trochochaetidae. *Bulletin of the United States National Museum*.
229. Pilsbry, H. A. (1917). Marine mollusks of Hawaii, IV-VII. *Proceedings of the Academy of natural sciences of Philadelphia*, 309-333.
230. Pilsbry, H. A. (1949). New Cerithiidae from Florida. *The Nautilus, 63*(2), 65-66.
231. Pilsbry, H. A., & McGinty, T. (1949). New marine mollusks of Florida and the Bahamas. *The Nautilus, 63*(1), 9-15.
232. Pilsbry, H. A., & McGinty, T. (1950). Vitrinellidae of Florida, Part 5. *The Nautilus, 63*(3), 85-87.
233. Pilsbury. (1893-1895). *Manual of conchology, structural and systematic, with illustrations of the species. Ser. 1. Vol. 15: Polyplacophora (Chitons). Acanthochitidae, Cryptoplacidae and appendix* (Vol. 15). Philadelphia: Conchological Section, Academy of Natural Sciences.
234. Pilsbury, H. (1917). Marine molluscs of Hawaii, IV-VII. *Proc. Acad. Nat. Sci. PhiIa, 69*.
235. Pimenta, A. D., Santos, F. N. D., & AbsalÃO, R. S. (2011). Taxonomic revision of the genus *Eulimella* (Gastropoda, Pyramidellidae) from Brazil, with description of three new species. *Zootaxa, 3063*(1), 22-38. doi:10.11646/zootaxa.3063.1.2
236. Pittman, C., & Fiene, P. (2023). Sea Slugs of Hawai’i. Retrieved from http://seaslugsofhawaii.com/
237. Pizzini, M., Nofroni, I., & Bonfitto, A. (2008). Two new species of Caecidae from the Indo-Pacific (Gastropoda). *Bollettino Malacologico, 44*(1/4), 21.
238. Pizzini, M., & Raines, B. (2011). The Caecidae from French Polynesia with description of eight new species (Caenogastropoda: Rissooidea). *Bollettino Malacologico, 47*(1), 23-46.
239. Pizzini, M., Raines, B. K., & Vannozzi, A. (2013). *The family Caecidae in the south-west Pacific (Gastropoda: Rissooidea)*: Società Italiana di Malacologia.
240. Platt, H., Warwick, R., & Furstenberg, J. P. (1985). Free-living marine nematodes. part 1 British Enoplids. In: Taylor & Francis.
241. Platt, H. M. (1982). *Revision of the Ethmolaimidae (Nematoda: Chromadorida)*: British Museum (Natural History).
242. Ponder, W. (1965a). The family Eatoniellidae in New Zealand. *Records of the Auckland Institute and Museum*, 47-99.
243. Ponder, W. (1965b). A revision of the New Zealand recent and fossil species of *Estea* Iredale, 1915. *Records of the Auckland Institute and Museum*, 131-159.
244. Ponder, W. (1968). Notes on New Zealand prosobranchs with descriptions of new species and subspecies. *Records of the Dominion Museum, 6*(8), 113-124.
245. Ponder, W., & Yoo, E. (1976). A revision of the Australian and tropical Indo-Pacific Tertiary and Recent species of Pisinna (= Estea)(Mollusca: Gastropoda: Rissoidae). *Rec. Aust. Mus, 30*, 150-247.
246. Ponder, W. F. (1999). Calopia (Calopiidae), a new genus and family of estuarine gastropods (Caenogastropoda: Rissooidea) from Australia. *Molluscan Research, 20*(1), 17-60. doi:10.1080/13235818.1999.10673722
247. Ponder, W. F., & Worsfold, T. (1994). *A Review of the Rissoiform Gastropods of Southwestern South America (Mollusca, Gastropoda)*: Natural History Museum of Los Angeles County.
248. Poppe, G. T., & Poppe, G. T. (2023). Conchology. Retrieved from https://www.conchology.be/
249. Poppe, G. T., Tagaro, S. P., & Stahlschmidt, P. (2015). New shelled molluscan species from the Central Philippines I. *Visaya, 4*(3), 15-59.
250. Poulin, R. (1995). Clutch size and egg size in free‐living and parasitic copepods: a comparative analysis. *Evolution, 49*(2), 325-336.
251. Powell, A. W. B. (1937). New species of marine Mollusca from New Zealand. *Discovery Reports, 15*, 153-222.
252. Powell, A. W. B. (1940, 1940). *The marine Mollusca of the Aupourian Province, New Zealand*.
253. Raines, B., & Pizzini, M. (2005). Contribution to the knowledge of the family Caecidae: 16. Revision of the Caecidae of Easter Island (Chile)(Caenogastropoda: Rissooidea Gray JE, 1847). *Iberus, 23*(1), 49-65.
254. Raines, B. K. (2019). Caecidae of the northeast Pacific (Gastropoda: Caenogastropoda). *Zoosymposia, 13*, 97–103-197–103.
255. Raines, B. K. (2020). A Rosetta Stone for eastern Pacific Caecidae (Gastropoda: Caenogastropoda). *Zootaxa, 4827*(1), 1–146-141–146.
256. Rato, J. Á. O., Gutiérrez, M. C., Abad, L. M., & Espinosa, J. (2014). What the shell tells in Aglajidae: a new genus for Aglaja felis (Opisthobranchia: Cephalaspidea). *Revista de la Academia Canaria de Ciencias:= Folia Canariensis Academiae Scientiarum, 26*(1), 83-119.
257. Raveendran, T., & Wagh, A. (1991). Distribution and growth of wood-borers in Bombay offshore waters.
258. Reeve, L. (1842). XXVI.—On the genus *Scarabus*, a small group of Pulmobranchiate Mollusks of the family Auriculacea. *Journal of Natural History, 9*(57), 218-221.
259. Rehder, H. A., & Ladd, H. S. (1973). Deep and shallow-water mollusks from the Central Pacific. *Science Reports of the Tohoku University, Sendai*, 37-49.
260. Renda, W., & Micali, P. (2016). *Vetulonia giacobbei* n. sp. from south Tyrrhenian Sea (Gastropoda, Seguenzioidea). *Bolletino Malacologico, 52*, 56-59.
261. Risbec, J. (1928). Contribution a l'etude des nudibranches Neo-Caledoniens. *Faune Colon. Franc., 2*, 3 maps.
262. Rolán, E., & Rubio, F. (2002). *The family Tornidae (Gastropoda, Rissooidea) in the East Atlantic* (Vol. 13): SEM.
263. Roper, C. F., Sweeney, M. J., & Nauen, C. (1984). Cephalopods of the world. An annotated and illustrated catalogue of species of interest to fisheries.
264. Rubio, F., & Rolán, E. (2013). Some new species of Skeneinae (Prosobranchia, Turbinidae). *Iberus, 31*, 1-9.
265. Rudman, W. (1971). On the opisthobranch genus *Haminoea* Turton & Kingston.
266. Salvador, R. B., & Cunha, C. M. (2016). Taxonomic revision of the fossil genera *Bulimactaeon,* *Hemiauricula (= Liocarenus)* and *Nucleopsis*, with description of a new Recent genus and species (Gastropoda: Heterobranchia: Acteonidae). *Journal of Molluscan studies, 82*(3), 472-483.
267. San Martín, G. (2005). Exogoninae (Polychaeta: Syllidae) from Australia with the description of a new genus and twenty-two new species. *Records of the Australian Museum, 57*(1), 39-152.
268. Sasaki, T. (2008). Micromolluscs in Japan: taxonomic composition, habitats, and future topics. *Zoosymposia, 1*, 147-232.
269. Saurin, E. (1958). Pyramidellidae de Pho-Hai (Sud Viet-Nam).
270. Saurin, E. (1959). Pyramidellidae de Nhatrang (Vietnam).
271. Saurin, E. (1961). Pyramidellidae du Golfe de Thailande. *Ann. Fac. Sci. Saigon, 1961*, 23-1266.
272. Schander, C. (1994). Twenty-eight new species of Pyramidellidae (Gastropoda, Heterobranchia) from west Africa. *Notiziario Cisma, 15*(1993), 11-78.
273. Schepman, M. M. (1913). *The Prosobranchia of the Siboga Expedition: Toxoglossa*: EJ Brill.
274. Schuurmans Stekhoven, J., Adam, W., & De Coninck, L. (1931). The freeliving marine nemas of the Belgian coast. *(No Title)*.
275. Sea Slug Forum. (2010). Retrieved from http://www.seaslugforum.net/
276. Smith, E. A. (1904a). I.—Natural History Notes from HM Indian Marine Survey Steamer ‘Investigator,’Commander TH Heming, RN—Series III., No. 1. On Mollusca from the Bay of Bengal and the Arabian Sea. *Journal of Natural History, 14*(79), 2-14.
277. Smith, E. A. (1904b). On a collection of marine shells from Port Alfred, Cape Colony. *Journal of Malacology, 11*(2), 21-44.
278. Smith, E. A. (1907). Mollusca. 2. Gastropoda. *National Antarctic Expedition 1901–1904. Natural History, 2*, 1-12.
279. Smith, E. A. (1910). *On South African marine Mollusca, with descriptions of new species*.
280. Smriglio, C., & Mariottini, P. (1996). Central Tyrrhenian Sea Mollusca: XI. Description of Callostracon tyrrhenicum sp. nov.(Gastropoda, Acteonidae) and remarks on the other Mediterranean species of the family Acteonidae d’Orbigny, 1835. *Basteria, 60*(4/6), 183-193.
281. Souverbie, S. M., & Montrouzier, X. (1865). Descriptions d'espèces nouvelles de l'Archipel Calédonien. *Journal de Conchyliologie, 13*, 150-158.
282. Sowerby, G. (1894). Descriptions of new species of marine shells from the neighbourhood of Hong-Kong. *Journal of Molluscan studies, 1*(4), 153-159.
283. Sowerby, G. (1900). Descriptions of new species of marine Mollusca collected by the late Otto Koch at the island of Cebu, Philippines. *Journal of Molluscan studies, 4*(3), 126-129.
284. Sowerby, G. B. (1892). *Marine shells of South Africa: a catalogue of all the known species*: Sowerby.
285. Sowerby, G. B. (1897). Appendix to Marine shells of South Africa: a catalogue of all the known species: with references to figures in various works, descriptions of new species, and figures of such as are new, little known, or hitherto unfigured.
286. Spurgeon, A. (2021). New Zeland Mollusca. Retrieved from https://www.mollusca.co.nz/
287. Stekhoven, J. H. S. (1950). *The Freeliving Marine Nemas of the Mediterranean*: Institut royal des sciences naturelles de Belgique.
288. Strong, E. E., & Bouchet, P. (2013). Cryptic yet colorful: anatomy and relationships of a new genus of Cerithiidae (Caenogastropoda, Cerithioidea) from coral reef drop‐offs. *Invertebrate Biology, 132*(4), 326-351.
289. Suter, H. (1898). Revision of the New Zealand Rissoiidae. *Journal of Molluscan studies, 3*(1), 2-8.
290. Tanamai, S., & Nabhitabhata, J. (2016). New records of marine cerithiid microgastropods (Prosobranchia: Cerithiidae) from Thai waters. *Phuket Marine Biological Center Research Bulletin, 73*, 1-5.
291. Tate, R., & May, W. (1900). Description of new genera and species of Australian Mollusca (chiefly Tasmanian). *Transactions of the Royal Society of South Australia, 24*(2), 90-103.
292. Taylor, J., & Walls, J. (1975). Cowries. In (pp. 288). New Jersey: TFH Publications.
293. Tenison-Woods, J. E. (1875). On some new Tasmanian marine shells. *Papers & Proceedings and Report of the Royal Society of Tasmania*, 131-159.
294. Tennson-Woods, J. E. (1874). Notes on the physical and zoological relations between Australia and Tasmania. *Monthly Notices of Papers & Proceedings of the Royal Society of Tasmania, 11*, 42-54. doi:10.1038/011091a0
295. Test, A. (1945). Description of new species of *Acmaea*. *Nautilus, 58*(3), 92-96.
296. Thiele, J. (1912). *Die antarktischen schnecken und muscheln.* Paper presented at the Deutsche Südpolar-Expedition, 1901-1903.
297. Thiele, J. (1925). Gastropoda der Deutschen Tiefsee-Expedition. II Teil. *Wiss. Ergebn. deutsche. Tiefsee-Expedition auf dem Dampher" Valdivia" 1898-1899, 17*(2), 35-382, pls. 313-346.
298. Timm, R. (1976). A redescription of the marine nematodes of Shackleton's British Antarctic expedition of 1907–1909. *Biology of the Antarctic Seas VI, 26*, 237-255.
299. Timm, R. W. (1952). A survey of the marine nematodes of Chesapeake Bay, Maryland.
300. Timm, R. W. (1976). Marine nematodes of the order Desmoscolecida from McMurdo Sound, Antarctica. *Antarctic Research Series*.
301. Treadwell, A. L. (1906). *Polychaetous annelids of the Hawaiian Islands collected by the steamer Albatross in 1902*: US Government Printing Office.
302. Treadwell, A. L. (1941). Polychaetous annelids from the New England region, Porto Rico and Brazil. American Museum novitates; no. 1138.
303. Turner, R. D. (2002). On the subfamily Xylophagainae (Family Pholadidae, Bivalvia, Mollusca). *Bulletin of the Museum of Compartive Zoology, 157*, 223-307.
304. Turton, W. H. (1932). The marine shells of Port Alfred, S. Africa. *(No Title)*.
305. Tuskes, P. M. (2019). Calliostomatidae of the northeast Pacific. *Zoosymposia, 13*, 83–96-83–96.
306. Tuskes, P. M., & Tuskes, A. (2019). Calliostoma and Akoya of the Californian Marine Province (Gastropoda: Calliostomatidae). *The Festivus, 35*, 11-28.
307. Valdés, Á. (2002). Review of the genus *Actinocyclus* Ehrenberg, 1831 (Ophisthobranchia: Doridoidea). *The Veliger, 45*(3), 193-202.
308. ValdÉs, Á., Lundsten, L., & Wilson, N. G. (2018). Five new deep-sea species of nudibranchs (Gastropoda: Heterobranchia: Cladobranchia) from the Northeast Pacific. *Zootaxa, 4526*(4), 401-433.
309. Van Aartsen, J. (2008). The Assimineidae of the Atlantic-Mediterranean seashores. *Basteria, 72*(4/6), 165-181.
310. Van der Linden, J., & Wagner, W. (1990). A key to the Recent European species of the genus *Bittium* Leach (Gastropoda, Prosobranchia, Cerithiidae). *Basteria, 54*(4/6), 243-246.
311. Vannozzi, A. (2016). Revision of the genus *Strebloceras* Carpenter, 1859 (Gastropoda: Caecidae). *Bolletino Malacologico, 52*, 110-121.
312. Vannozzi, A., Pizzini, M., & Raines, B. (2015). Revision of South African Caecidae (Mollusca: Gastropoda). *African Invertebrates, 56*(1), 99-136.
313. Vélain, C. (1887). Laboratoire de géologie à la Faculté des Sciences. *Annuaires de l'École pratique des hautes études, 3*(1), 168-182.
314. Venkataraman, K. (2013). *Free-living Marine nematodes of Tamil Nadu Coast, India*: Zoological Survey of India.
315. Verco, J. C. (1904). *Notes on South Australian Marine Mollusca with Descriptions of New Species. Part I [-XIV]*.
316. Verco, J. C. (1909). Notes on South Australian Marine Mollusca with Descriptions of New Species. Part VII. *Transactions of the Royal Society of South Australia, 31*, 305-315.
317. Verhecken, A. (2011). *The Cancellariidae of the PANGLAO Marine Biodiversity Project 2004 and the PANGLAO 2005 and AURORA 2007 deep sea cruises in the Philippines, with description of six new species (Neogastropoda, Cancellarioidea)*: Nederlandse Malacologische Vereniging.
318. Vilvens, C. (2005). New records and new species of *Calliostoma* and *Bathyfaiitor* (Gastropoda: Calliostomatidae) from the Vanuatu, Fiji and Tonga.
319. Vilvens, C. (2012). New species and new records of Seguenzioidea and Trochoidea (Gastropoda) from French Polynesia. *Novapex, 13*, 1-23.
320. Vilvens, C. (2014). New species and new records of Calliostomatidae (Gastropoda: Trochoidea).
321. Warén, A. (1989). New and little known Mollusca from Iceland. *Sarsia, 74*(1), 1-28.
322. Warén, A. (1992). New and little known" skeneimorph" gastropods from the Mediterranean Sea and the Atlantic Ocean. *Boll. Malac., 27*, 149-247.
323. Warén, A. (1993). New and little-known mollusca from Iceland and Scandinavia. Part 2. *Sarsia, 78*(3-4), 159-201.
324. Warén, A. (1996). New and little known Mollusca from Iceland and Scandinavia. Part 3. *Sarsia, 81*(3), 197-245.
325. Warén, A., & Bouchet, P. (1993). New records, species, genera, and a new family of gastropods from hydrothermal vents and hydrocarbon seeps. *Zoologica Scripta, 22*(1), 1-90.
326. Watson, R. B. (1879). Mollusca of HMS ‘Challenger’Expedition. II. The Solenoconchia, comprising the Genera Dentalium, Siphodentalium, and Cadulus. *Journal of the Linnean Society of London, Zoology, 14*(78), 508-529.
327. Weiser, W. (1956). *Free-living Marine Nematodes III. Axonolaimoidea and Monhysteroidea*: Lund Hakan Oilssons Boktrycker.
328. Węsławski, J. M., Legeżyńska, J., & Włodarska‐Kowalczuk, M. (2020). Will shrinking body size and increasing species diversity of crustaceans follow the warming of the Arctic littoral? *Ecology and Evolution, 10*(19), 10305-10313.
329. White, K. M. (1946). On a new species of Aglaja from Ceylon. *Journal of Molluscan studies, 26*(6), 167-172.
330. Wieser, W. (1953). Free-living marine nematodes. I. Enoploidea. Chile reports 10. *Lund. Univ. Arsskrift, 49*, 1-155.
331. Wieser, W. (1954). Free-living marine nematodes II. Chromadoroidea. *Acta Universitatis Lundensis NF, 2*(50), 1-148.
332. Wilson, B. (1994). *Australian Marine Shells (Vol. 1 2)*: Australia: Odyssey; ISBN 0 646 15226 2 ISBN 0 646 15227 0.
333. Yidi Daccarett, E., & Bossio, V. S. (2011). Colombian seashells from the Caribbean Sea. *LInformatore Piceno, Anacona, Italia*.
334. Yokoyama, M. (1922). Fossils from the Upper Mussashino of Kazusa and Shimosa. *44*, p-1.
335. Yonow, N. (1994). *Opisthobranchs from the Maldive Islands, including descriptions of seven new species (Mollusca: Gastropoda)*: Musée de zoologie.
336. Yonow, N., & Jensen, K. R. (2018). Results of the Rumphius Biohistorical Expedition to Ambon (1990). Part 17. The Cephalaspidea, Anaspidea, Pleurobranchida, and Sacoglossa (Mollusca: Gastropoda: Heterobranchia). *Archiv für Molluskenkunde International Journal of Malacology, 147*(1), 1-48. doi:10.1127/arch.moll/147/001-048
337. Zelaya, D. G., Schejter, L., & Ituarte, C. (2011). *Neactaeonina argentina*, new Species, and family placement of the genus *Neactaeonina* Thiele, 1912 (Mollusca: Gastropoda). *Malacologia, 53*(2), 251-263. doi:10.4002/040.053.0204
