# Supplementary material for: Navigating uncertainty in maximum body size in marine metazoans
Source: Ecol Evol. 2024 Jun 5;14(6):e11506. doi: 10.1002/ece3.11506 (PMC11151150; doi:10.1002/ece3.11506)
Supplement: Supplementary file 2 — Appendix S2. [file ECE3-14-e11506-s002.docx]

## SUPPLEMENTAL

**Supplemental Table 1:** Compute estimated marginal means (EMMs) for specified factors and contrasts among them for phylum.

| **Phylum** | **Emmean** | **SE** | **DF** | **Asymp. LCL** | **Asymp. UCL** |
| --- | --- | --- | --- | --- | --- |
| Annelida | -0.601 | 0.075 | Inf | -0.748 | -0.455 |
| Arthropoda | -1.854 | 0.034 | Inf | -1.920 | -1.788 |
| Bryozoa | -1.358 | 0.060 | Inf | -1.476 | -1.240 |
| Chordata | -1.451 | 0.024 | Inf | -1.498 | -1.404 |
| Cnidaria | -0.804 | 0.061 | Inf | -0.923 | -0.685 |
| Echinodermata | -0.712 | 0.067 | Inf | -0.844 | -0.580 |
| Mollusca | -1.437 | 0.021 | Inf | -1.478 | -1.395 |
| Nematoda | -2.394 | 0.119 | Inf | -2.627 | -2.161 |

| **Contrast** | **Estimate** | **SE** | **DF** | **z-ratio** | **p-value** |
| --- | --- | --- | --- | --- | --- |
| Annelida - Arthropoda | 1.253 | 0.079 | Inf | 15.827 | <.0001 |
| Annelida - Bryozoa | 0.757 | 0.094 | Inf | 8.087 | <.0001 |
| Annelida - Chordata | 0.850 | 0.075 | Inf | 11.395 | <.0001 |
| Annelida - Cnidaria | 0.203 | 0.094 | Inf | 2.165 | 0.3732 |
| Annelida - Echinodermata | 0.111 | 0.097 | Inf | 1.140 | 0.9481 |
| Annelida - Mollusca | 0.835 | 0.074 | Inf | 11.306 | <.0001 |
| Annelida - Nematoda | 1.793 | 0.138 | Inf | 12.990 | <.0001 |
| Arthropoda - Bryozoa | -0.496 | 0.066 | Inf | -7.486 | <.0001 |
| Arthropoda - Chordata | -0.403 | 0.035 | Inf | -11.515 | <.0001 |
| Arthropoda - Cnidaria | -1.049 | 0.067 | Inf | -15.764 | <.0001 |
| Arthropoda - Echinodermata | -1.142 | 0.072 | Inf | -15.856 | <.0001 |
| Arthropoda - Mollusca | -0.417 | 0.034 | Inf | -12.345 | <.0001 |
| Arthropoda - Nematoda | 0.540 | 0.121 | Inf | 4.454 | 0.0002 |
| Bryozoa - Chordata | 0.093 | 0.061 | Inf | 1.523 | 0.7954 |
| Bryozoa - Cnidaria | -0.554 | 0.084 | Inf | -6.631 | <.0001 |
| Bryozoa - Echinodermata | -0.646 | 0.088 | Inf | -7.379 | <.0001 |
| Bryozoa - Mollusca | 0.079 | 0.060 | Inf | 1.309 | 0.8958 |
| Bryozoa - Nematoda | 1.036 | 0.132 | Inf | 7.849 | <.0001 |
| Chordata - Cnidaria | -0.647 | 0.061 | Inf | -10.634 | <.0001 |
| Chordata - Echinodermata | -0.739 | 0.067 | Inf | -11.072 | <.0001 |
| Chordata - Mollusca | -0.015 | 0.021 | Inf | -0.700 | 0.997 |
| Chordata - Nematoda | 0.943 | 0.119 | Inf | 7.930 | <.0001 |
| Cnidaria - Echinodermata | -0.092 | 0.088 | Inf | -1.051 | 0.9665 |
| Cnidaria - Mollusca | 0.632 | 0.061 | Inf | 10.448 | <.0001 |
| Cnidaria - Nematoda | 1.590 | 0.132 | Inf | 12.077 | <.0001 |
| Echinodermata - Mollusca | 0.725 | 0.066 | Inf | 10.972 | <.0001 |
| Echinodermata - Nematoda | 1.682 | 0.134 | Inf | 12.540 | <.0001 |
| Mollusca - Nematoda | 0.957 | 0.118 | Inf | 8.102 | <.0001 |

**Supplemental Table 2:** Compute estimated marginal means (EMMs) for specified factors and contrasts among them for habitat.

| **Phylum** | **Emmean** | **SE** | **DF** | **Asymp. LCL** | **Asymp. UCL** |
| --- | --- | --- | --- | --- | --- |
| benthic | -1.34 | 0.02 | Inf | -1.39 | -1.29 |
| pelagic | -1.62 | 0.03 | Inf | -1.67 | -1.557 |
| unspecified | -1.03 | 0.04 | Inf | -1.11 | -0.942 |

| **Contrast** | **Estimate** | **SE** | **DF** | **z-ratio** | **p-value** |
| --- | --- | --- | --- | --- | --- |
| benthic -pelagic | 0.277 | 0.0185 | Inf | 14.946 | <.0001 |
| benthic -unspecified | -0.313 | 0.0358 | Inf | -8.758 | <.0001 |
| pelagic -unspecified | -0.59 | 0.0382 | Inf | -15.451 | <.0001 |

**Supplemental Figure 1:** Consequences of randomly selecting a single body size value for each species on overall rank order of species body sizes across all 27,581 species, based on 1000 randomizations. The correlation between two independent rankings of body size based on randomly selecting a single size value for each species was always high, averaging 0.981 (**A**). The average species shifts rank by only about 24 positions in the full body size rank order between any pairs of random draws of a single size value per species (B), however in any one of the 1000 randomizations, the species with the largest shift in rankings typically moves almost from one end of the ranking to the other, with a mean shift of over 25,000 places (**C**).

**Supplemental Table 3:** Species with maxsize_range_ > 2, indicating >2 orders of magnitude difference between smallest and largest maximum size (linear, cm). Also shows is the number of times (out of 1000 randomisations) that each species in this list had the largest change in body size rank order across all 27,571 species when randomly selecting single estimates of maximum size per species.

| **Phylum** | **class** | **scientificName** | **AphiaID** | **Smallest maximum size (cm)** | **Largest maximum size (cm)** | **Maxsize­_range_** | **N(max rank shift)** | **Explanation for large Maxsize­_range_** |
| --- | --- | --- | --- | --- | --- | --- | --- | --- |
| Arthropoda | Ostracoda | *Asteropteron fuscum* | 451024 | 0.000053 | 0.177 | 3.52 | - | Error in reported measurement |
| Bryozoa | Gymnolaemata | *Rosseliana rosselii* | 111431 | 0.030 | 71.5 | 3.38 | 303 | Comparing colony size with individual zooid size |
| Bryozoa | Gymnolaemata | *Bugula neritina* | 111158 | 0.030 | 69.1 | 3.36 | 40 | Comparing colony size with individual zooid size |
| Annelida | Polychaeta | *Polyophthalmus mauliola* | 1326881 | 0.25 | 518 | 3.31 | 6 | Error of measurement units |
| Bryozoa | Gymnolaemata | *Electra monostachys* | 111354 | 0.028 | 54.8 | 3.29 | 16 | Comparing colony size with individual zooid size |
| Bryozoa | Gymnolaemata | *Flustra foliacea* | 111367 | 0.028 | 52.3 | 3.27 | 9 | Comparing colony size with individual zooid size |
| Bryozoa | Gymnolaemata | *Conopeum reticulum* | 111351 | 0.030 | 50.1 | 3.22 | 5 | Comparing colony size with individual zooid size |
| Bryozoa | Gymnolaemata | *Electra pilosa* | 111355 | 0.035 | 58.4 | 3.22 | 23 | Comparing colony size with individual zooid size |
| Bryozoa | Gymnolaemata | *Conopeum seurati* | 111352 | 0.040 | 65.5 | 3.21 | 159 | Comparing colony size with individual zooid size |
| Bryozoa | Gymnolaemata | *Megapora ringens* | 111217 | 0.030 | 47.6 | 3.20 | 7 | Comparing colony size with individual zooid size |
| Bryozoa | Gymnolaemata | *Calpensia nobilis* | 111425 | 0.040 | 59.4 | 3.17 | 44 | Comparing colony size with individual zooid size |
| Mollusca | Bivalvia | *Bankia setacea* | 527858 | 0.70 | 1,000 | 3.15 | - | Comparing shell size with length of foot |
| Annelida | Polychaeta | *Westheidesyllis heterocirrata* | 761621 | 0.17 | 218 | 3.12 | 76 | Comparing width and length dimensions |
| Bryozoa | Gymnolaemata | *Beania mirabilis* | 111072 | 0.065 | 82.3 | 3.10 | 223 | Comparing colony size with individual zooid size |
| Arthropoda | Ostracoda | *Porroecia pseudoparthenoda* | 127855 | 0.00017 | 0.190 | 3.04 | - | Error in measurement |
| Bryozoa | Gymnolaemata | *Cribrilina punctata* | 111314 | 0.045 | 47.4 | 3.02 | 1 | Comparing colony size with individual zooid size |
| Annelida | Polychaeta | *Sclerolinum brattstromi* | 129129 | 0.013 | 13.4 | 3.01 | - | Comparing width and length dimensions |
| Bryozoa | Gymnolaemata | *Cribrilina (Cribrilina) cryptooecium* | 1059487 | 0.055 | 48.2 | 2.94 | 1 | Comparing colony size with individual zooid size |
| Echinodermata | Holothuroidea | *Oestergrenia digitata* | 152547 | 0.035 | 30.0 | 2.93 | - | Comparing disc diameter with arm length |
| Bryozoa | Gymnolaemata | *Carbasea carbasea* | 111362 | 0.12 | 96.7 | 2.91 | 85 | Comparing colony size with individual zooid size |
| Echinodermata | Asteroidea | *Asterias rubens* | 123776 | 0.098 | 60.0 | 2.79 | 1 | Comparing disc diameter with arm length |
| Mollusca | Bivalvia | *Teredo bartschi* | 141605 | 0.40 | 230 | 2.76 | - | Comparing shell size with length of foot |
| Mollusca | Bivalvia | *Lyrodus pedicellatus* | 141600 | 0.40 | 205 | 2.71 | - | Comparing shell size with length of foot |
| Mollusca | Bivalvia | *Bankia carinata* | 141598 | 0.40 | 191 | 2.68 | - | Comparing shell size with length of foot |
| Cnidaria | Hydrozoa | *Praya dubia* | 135466 | 10 | 4,570 | 2.66 | - | Natural intraspecific variation in maximum size |
| Cnidaria | Hydrozoa | *Pennaria disticha* | 117802 | 0.070 | 30.0 | 2.63 | - | Natural intraspecific variation in maximum size |
| Bryozoa | Gymnolaemata | *Crisularia plumosa* | 834039 | 0.020 | 8.00 | 2.60 | - | Comparing colony size with individual zooid size |
| Bryozoa | Gymnolaemata | *Crisularia purpurotincta* | 834041 | 0.020 | 8.00 | 2.60 | - | Comparing colony size with individual zooid size |
| Cnidaria | Hydrozoa | *Velella velella* | 117832 | 0.030 | 10.0 | 2.52 | - | Error in reported measurement |
| Bryozoa | Gymnolaemata | *Bugulina turbinata* | 834020 | 0.020 | 6.00 | 2.48 | - | Comparing colony size with individual zooid size |
| Chordata | Ascidiacea | *Polysyncraton lacazei* | 103593 | 0.0020 | 0.500 | 2.40 | - | Comparing colony size with individual zooid size |
| Cnidaria | Anthozoa | *Platygyra daedalea* | 207489 | 0.60 | 100 | 2.22 | - | Comparing colony size with individual zooid size |
| Mollusca | Bivalvia | *Bankia bipalmulata* | 527846 | 1.2 | 175 | 2.18 | - | Comparing shell size with length of foot |
| Cnidaria | Anthozoa | *Virgularia mirabilis* | 128539 | 0.40 | 60.0 | 2.18 | - | Comparing colony size with individual zooid size |
| Bryozoa | Gymnolaemata | *Bugulina fulva* | 834010 | 0.020 | 3.00 | 2.18 | - | Comparing colony size with individual zooid size |
| Bryozoa | Gymnolaemata | *Bugulina simplex* | 834016 | 0.020 | 3.00 | 2.18 | - | Comparing colony size with individual zooid size |
| Annelida | Polychaeta | *Polygordius appendiculatus* | 130712 | 0.031 | 4.50 | 2.16 | - | Comparing width and length dimensions |
| Bryozoa | Gymnolaemata | *Bugulina stolonifera* | 834018 | 0.030 | 4.00 | 2.12 | - | Comparing colony size with individual zooid size |
| Annelida | Polychaeta | *Arabella iricolor* | 129854 | 0.50 | 60.0 | 2.08 | - | Comparing width and length dimensions |
| Annelida | Polychaeta | *Lanice conchilega* | 131495 | 0.25 | 30.0 | 2.08 | - | Comparing width and length dimensions |
| Annelida | Polychaeta | *Lumbrineris coccinea* | 130242 | 0.15 | 17.0 | 2.05 | - | Comparing width and length dimensions |
| Chordata | Ascidiacea | *Leptoclinides madara* | 253135 | 0.0090 | 1.00 | 2.05 | - | Comparing colony size with individual zooid size |
| Chordata | Teleostei | *Pleuronectes platessa* | 127143 | 1.1 | 122 | 2.03 | - | Comparing adult and juvenile |
| Chordata | Mammalia | *Lagenodelphis hosei* | 137099 | 2.6 | 270 | 2.02 | - | Error or measurement units |
